# Ancestral polymorphisms explain the role of chromosomal inversions in speciation

**DOI:** 10.1371/journal.pgen.1007526

**Published:** 2018-07-30

**Authors:** Zachary L. Fuller, Christopher J. Leonard, Randee E. Young, Stephen W. Schaeffer, Nitin Phadnis

**Affiliations:** 1 Department of Biology, Erwin W. Mueller Laboratories, The Pennsylvania State University, University Park, PA, United States of America; 2 Department of Biology, University of Utah, Salt Lake City, UT, United States of America; University of Michigan, UNITED STATES

## Abstract

Understanding the role of chromosomal inversions in speciation is a fundamental problem in evolutionary genetics. Here, we perform a comprehensive reconstruction of the evolutionary histories of the chromosomal inversions in *Drosophila persimilis* and *D*. *pseudoobscura*. We provide a solution to the puzzling origins of the selfish *Sex-Ratio* arrangement in *D*. *persimilis* and uncover surprising patterns of phylogenetic discordance on this chromosome. These patterns show that, contrary to widely held views, all fixed chromosomal inversions between *D*. *persimilis* and *D*. *pseudoobscura* were already present in their ancestral population long before the species split. Our results suggest that patterns of higher genomic divergence and an association of reproductive isolation genes with chromosomal inversions may be a direct consequence of incomplete lineage sorting of ancestral polymorphisms. These findings force a reconsideration of the role of chromosomal inversions in speciation, not as protectors of existing hybrid incompatibilities, but as fertile grounds for their formation.

## Author summary

Studies on chromosomal inversions and reproductive isolation between *Drosophila persimilis* and *D*. *pseudoobscura* have played a profound role in shaping our understanding of inversions, speciation and selfish chromosomes. In this study, we reconstruct the evolutionary histories of chromosomal inversions in *D*. *persimilis* and *D*. *pseudoobscura* to show that, contrary to widely accepted ideas, these inversions existed as polymorphisms in the ancestor of both species before their initial split. These findings force a reconsideration of the role of chromosomal inversions in speciation and raise the possibility that the higher genetic divergence of sequences spanning these chromosomal inversions and an association with hybrid incompatibility genes may be an emergent property of the long-term segregation of these inversions.

## Introduction

Chromosomal inversions are structural rearrangements where the linear gene order is reversed. In crosses between two species that differ by one or more inversions, the resulting hybrids can experience meiotic chromosome pairing problems and may, therefore, become sterile. Chromosomal inversions can, thus, potentially play an important role in the evolution of intrinsic postzygotic barriers between species. Understanding the extent to which such chromosomal rearrangements play a role in speciation is a longstanding and fundamental problem in evolutionary genetics [1–3]. In a number of plant species, direct experimental evidence has cemented the role of chromosomal rearrangements in the evolution of reproductive isolation through the reduced fertility in heterokaryotic hybrids [1,4–6]. In contrast, classic studies in hybrids between *Drosophila persimilis* and *D*. *pseudoobscura* have shown that chromosomal inversions do not play a direct role in causing hybrid sterility in animal species [2,4]. There is now clear evidence for genic incompatibilities as the cause hybrid sterility in many cases, and the idea that chromosomal inversions may play a role in animal speciation fell out of favor [2,4]. Recent studies in *D*. *persimilis* and *D*. *pseudoobscura*–the same species that helped lead to the demise of the idea of a direct role of chromosomal inversions in hybrid sterility–,however, have led to a dramatic resurgence of a modified version for the role of chromosomal inversions in speciation. Two new empirical observations regarding the patterns of reproductive isolation and genetic divergence in *D*. *persimilis* and *D*. *pseudoobscura* are key to these developments: i) the fixed chromosomal inversions between these species display higher genetic divergence than collinear regions of the genome, and ii) nearly all genes that contribute to reproductive isolation between these species are located among the fixed chromosomal inversion differences [7–12].

These two empirical patterns are explained by new versions of the chromosomal theory of speciation, which may be explained as follows. Consider a single species that has recently separated into two isolated populations [9,13]. These populations evolve independently, and genes that contribute to reproductive isolation initially evolve uniformly across the genome in both populations. If these populations were to later re-hybridize on secondary contact, any incompatible alleles will be selected against because such alleles suffer a fitness cost in the form of unfit hybrid progeny. However, in populations that have evolved fixed inversions differences, incompatible alleles may become locked together with beneficial alleles in large blocks of tightly linked loci generated by the recombination suppressing properties of inversions. In such a situation, linked beneficial alleles may prevent selection from eliminating the incompatible alleles on secondary contact and, thus, help maintain reproductive isolation between these incipient species during secondary contact.

In contrast, collinear regions of the genome may continue to exchange genes, leading to the elimination of incompatible alleles in these regions. Any gene flow between species is, thus, prevented within genomic regions spanning chromosomal inversions through the maintenance of hybrid incompatibilities, but continues across collinear regions of the genomes. Due to this heterogeneous pattern of gene flow across the genome after the initial evolution of reproductive isolating barriers, hybrid incompatibility alleles may become disproportionately associated with chromosomal inversion differences between species, and genomic regions spanning inversions may appear more genetically diverged as compared to collinear regions. This ‘speciation with gene flow’ process can, thus, explain both empirical patterns found in *Drosophila persimilis* and *D*. *pseudoobscura*, which represent one of the most thoroughly studied hybridizations in speciation genetics. Consistent with this idea, Dobzhansky (1973) observed a single hybrid female between *D*. *pseudoobscura* and *D*. *persimilis* from nature and multiple studies have detected genomic signatures of recent gene flow between these species, suggesting that these species may continue to exchange genes at a detectable level [12,14–17]. Moreover, the empirical patterns described above appear difficult to explain without invoking a major role for gene flow after the initial evolution of reproductive isolation. Together, these results support the ‘speciation with gene flow’ idea, and have led to the widespread acceptance of the role of recombination suppression by chromosomal inversions in the maintenance of animal species [11,18–23].

Here, we comprehensively dissect the evolutionary histories of the chromosomal inversions in *D*. *persimilis* and *D*. *pseudoobscura* to show that, contrary to the currently accepted view, all fixed chromosomal inversions between these species segregated in their common ancestral population, and pre-dated the divergence between these species by a remarkable length of time. Our key insights into deciphering the evolutionary histories of these chromosomal inversions came from resolving the origins of the chromosomal arrangement associated with the *D*. *persimilis* Sex-Ratio phenotype and from uncovering strong patterns of phylogenetic discordance along the *Sex-Ratio* chromosome. We, therefore, explain our resolution of the evolutionary history of this *Sex-Ratio* chromosome before proceeding to reconstruct the evolutionary history of the fixed chromosomal inversions differences between *D*. *persimilis* and *D*. *pseudoobscura*.

**Fig 1:**
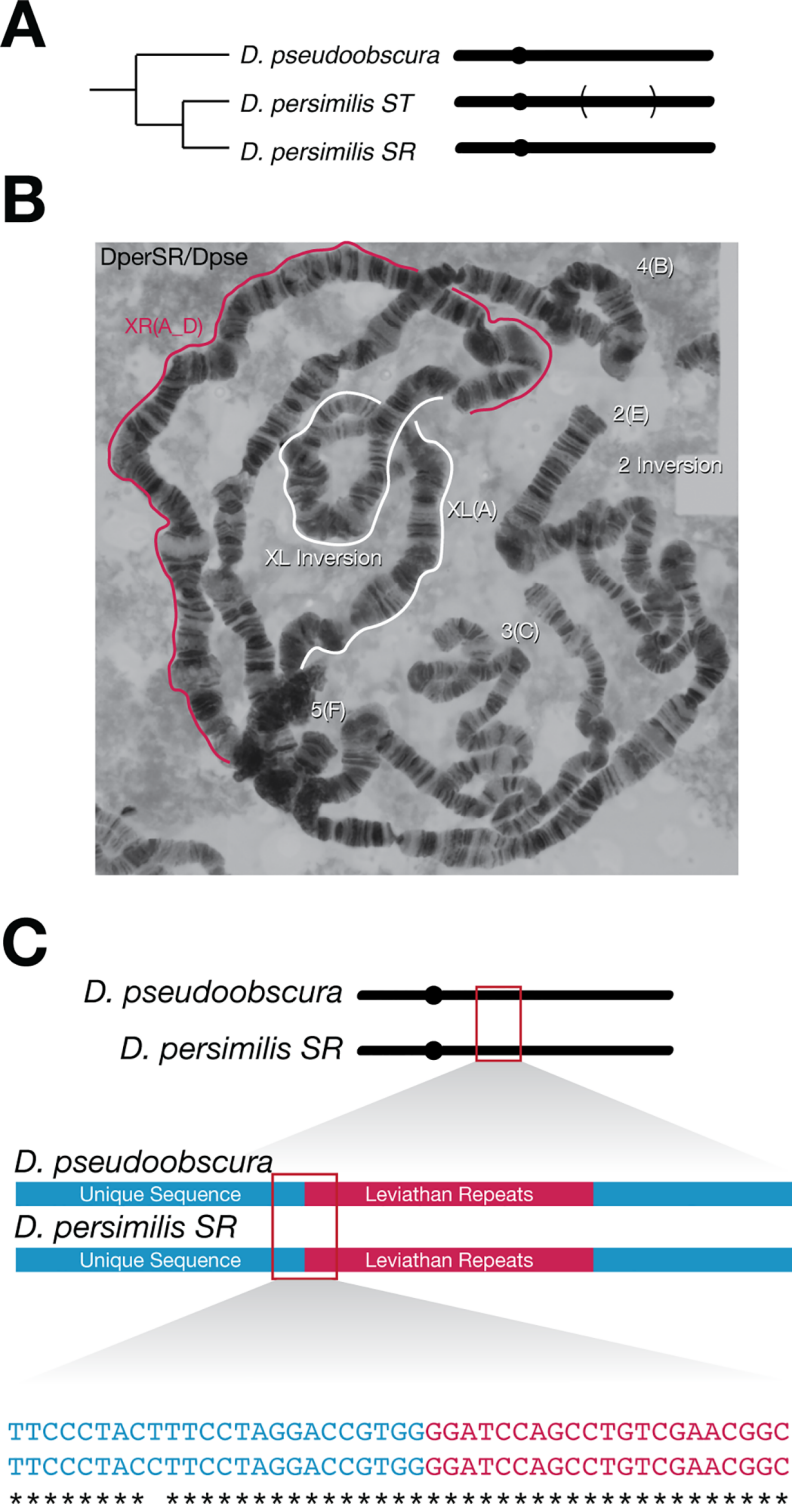
The *D*. *persimilis Sex-Ratio* (*SR*) chromosome is precisely collinear with *D*. *pseudoobscura*. *(A)* The right arm of the *X* chromosome (*XR*) of *D*. *persimilis* is normally inverted as compared to its sister species, *D*. *pseudoobscura*, but the *D*. *persimilis Sex-Ratio* chromosome is collinear with its sister species. (B) Polytene chromosome squash of a *D*. *persimilis SR/D*. *pseudoobscura* hybrid female demonstrating perfect interspecies collinearity on *XR*. (C) Amplification and sequencing of the proximal breakpoint of the *D*. *persimilis* inversion reveals that the breakpoints are collinear at the base-pair level.

*Sex-Ratio* chromosomes are variants of *X*-chromosomes that are often found at high frequencies within natural populations [24]. Males that carry a *Sex-Ratio* chromosome eliminate nearly all *Y*-bearing sperm [25], and produce nearly all female offspring (*i*.*e*., heavily distorted progeny *sex-ratios*). By distorting the balance of segregation in their favor in excess of Mendelian expectations, these *Sex-Ratio* chromosomes can rapidly spread through populations even if they reduce the fitness of the individuals that carry them [26,27]. When a new chromosomal inversion generates tight linkage between an existing segregation distorter allele and other alleles that enhance distortion (or alleles that neutralize suppressors-of-distortion), this produces a stronger driving chromosome that can supplant its weaker versions [28]. This process sets up an expected order for the evolution of *Sex-Ratio* chromosomes: distorter alleles arise first, enhancers of distortion appear next, and chromosomal inversions that tie these together arrive last. This framework explains why most *Sex-Ratio* chromosomes are associated with derived inversions relative to the wild type, or *Standard* (*ST*) chromosomes [27]. Consistent with this pattern, the *D*. *persimilis SR* chromosome is inverted with respect to the *D*. *persimilis ST* chromosome on the right arm of the *X* chromosome (*XR*). However, the *Standard D*. *persimilis XR* differs from *D*. *pseudoobscura XR* by a single derived inversion. Curiously, the *D*. *persimilis SR* inversion appears to have reversed the same derived *D*. *persimilis ST* inversion, such that *D*. *persimilis SR* appears collinear with *D*. *pseudoobscura* (Fig 1A). It is not clear whether this unexpected collinearity of the *D*. *persimilis SR* chromosome with the *ST* chromosome of its sister species is the result of a second inversion event on the background of *D*. *persimilis ST* at approximately the same breakpoints as the original *D*. *persimilis XR* inversion, or whether a single chromosomal arrangement was inherited from the ancestor of the two species [29,30]. Previous molecular evolutionary studies on the origins of this chromosome have yielded conflicting results, and the origin of the *D*. *persimilis Sex-Ratio* inversion remains the subject of speculation [7,31,32].

Here, we show that the *D*. *persimilis SR* chromosome did not arise from a second inversion event, but is the ancestrally-arranged chromosome. Surprisingly, we also discovered large blocks of phylogenetic discordance in the regions flanking the *D*. *persimilis SR* inversion breakpoints, such that they are more closely related to the *D*. *pseudoobscura*, rather than to the *D*. *persimilis ST* chromosome. These patterns provide evidence that, contrary to the currently held view, fixed rearrangement differences between *D*. *persimilis* and *D*. *pseudoobscura* arose in the ancestor of the two species before being passed exclusively to *D*. *persimilis*. Using whole-genome data in this same model system, Kulathinal et al. (2009) concluded that the similarly observed patterns of increased divergence associated with inverted regions was the result of *D*. *persimilis* having acquired all three inversions after speciation and the homogenizing effect of post-secondary contact. In our study, with higher resolution sequences, multiple statistical approaches, and the inclusion of the *D*. *persimilis* SR arrangement, we instead show that all fixed inversion differences are the result of ancestrally segregating polymorphisms and offer a model which does not rely on post-speciation gene flow or ongoing hybridization to explain the observed patterns of divergence. Together, our results challenge our current understanding of the evolutionary history of the inversions in *D*. *persimilis* and *D*. *pseudoobscura*, and suggest that ancestrally segregating polymorphisms may play a critical role in establishing the patterns of divergence and an association of reproductive isolation genes with chromosomal inversion differences between species.

## Results

### *D*. *pseudoobscura XR* and *D*. *persimilis Sex-Ratio* are precisely collinear

We isolated two independent *D*. *persimilis SR* strains that produce >90% female progeny, and generated high quality mosaic images of polytene chromosomes from squashes of larval salivary glands. Consistent with previous reports [24], the *D*. *persimilis SR* chromosome differs by one major inversion on *XR* with respect to *D*. *persimilis ST*, but appears collinear with *D*. *pseudoobscura* (Fig 1B, S1 Fig). If *D*. *persimilis SR* was derived from *D*. *persimilis ST* through a somewhat imprecise reversion to the ancestral arrangement, the banding patterns of polytene chromosomes in hybrid *D*. *persimilis SR*/*D*. *pseudoobscura* females may reveal slight imperfections near the inversion breakpoints. We did not observe any disruption of chromosome pairing near the inversion breakpoints in *D*. *persimilis SR/D*. *pseudoobscura* heterozygotes, suggesting that any secondary inversion event may have been in close proximity to the original breakpoints of the *D*. *persimilis ST* inversion.

While our polytene analyses showed no visible aberrations at the breakpoints of the *D*. *persimilis* inversion, such analyses provide only a coarse view of chromosome structure. Previously, the *D*. *persimilis ST* inversions breakpoints were mapped at a resolution of 30kb [10]. To precisely identify the inversion breakpoints on the *D*. *persimilis SR* chromosome, we first performed whole genome sequencing of males pooled from two *D*. *persimilis SR* strains, as well as males pooled from two *D*. *persimilis ST* strains. Using the approximate genomic coordinates of the inversion breakpoints, we designed multiple primer pairs that span the proximal and distal inversion breakpoint sequences from *D*. *persimilis SR* and *D*. *pseudoobscura*. We were able to successfully amplify sequences corresponding to the proximal breakpoint (S2 Fig) and Sanger sequencing of these products revealed the presence of four 319bp *Leviathan* repeats [33]. More importantly, *D*. *persimilis SR* and *D*. *pseudoobscura* sequences that flank the *Leviathan* repeats are precisely collinear to a single base pair resolution (Fig 1C). We were unable to amplify the sequences across the distal breakpoint, likely because of the presence of a large block of repetitive sequences accumulated at this breakpoint after the initial inversion event. Nevertheless, information about the proximal inversion breakpoint accurately provides the position of the distal breakpoint, which is sufficient to answer the questions that we seek to address here. In particular, our results from the proximal breakpoint show that a slightly staggered second inversion event is not the basis for the collinearity between the *D*. *persimilis SR* and *D*. *pseudoobscura* chromosomes.

### The *D*. *persimilis Sex-Ratio* chromosome is more closely related to *D*. *pseudoobscura* than to *D*. *persimilis* at the inversion breakpoints

**Fig 2:**
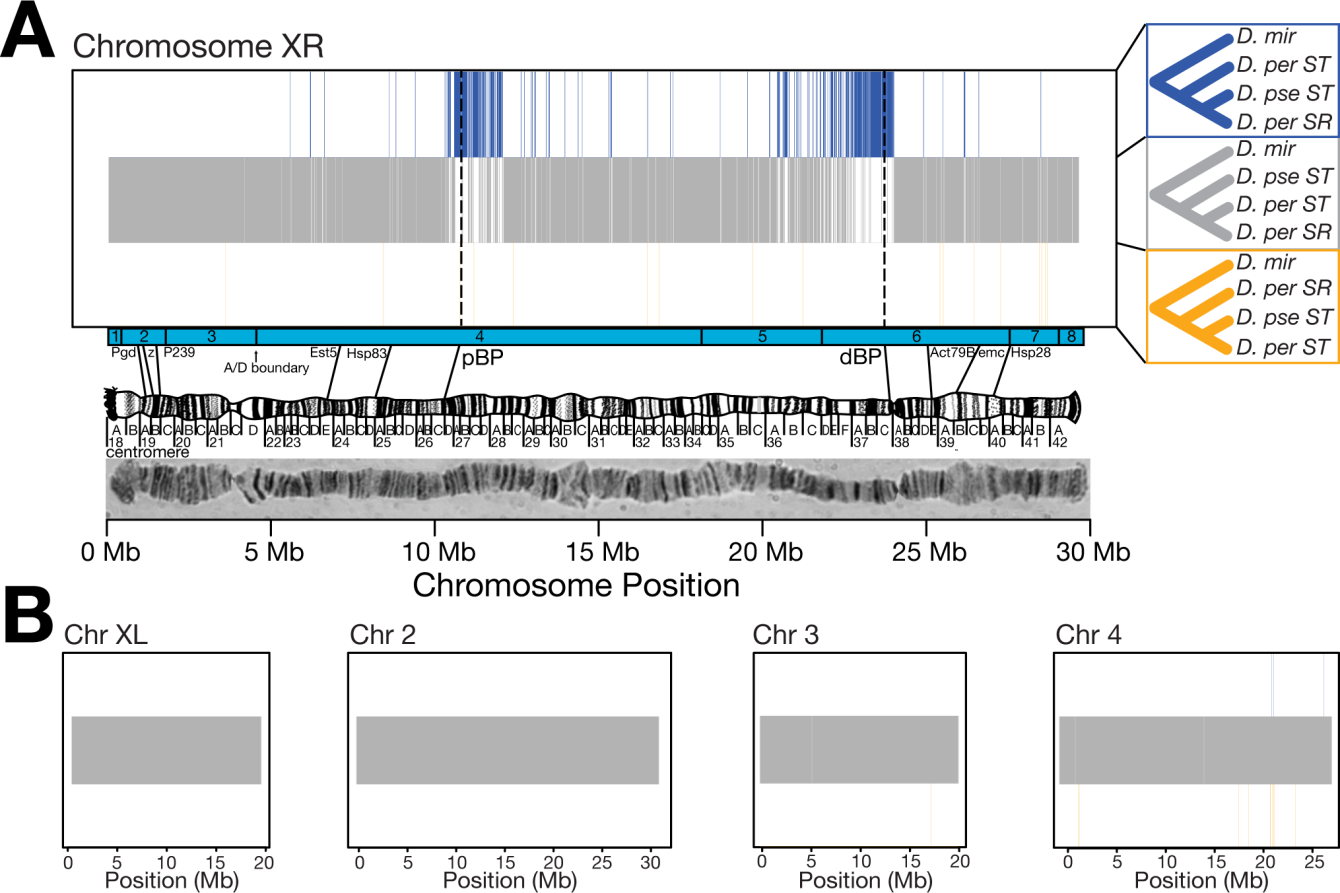
The inversion breakpoints on *XR* show extensive phylogenetic discordance. *(A)* Sliding window phylogeny classification on *XR*. Blue, grey, and orange vertical lines represent the tree topology supported by neighbor-joining trees. Grey trees represent no phylogenetic discordance. Blue trees represent regions where the two collinear chromosomes appear more similar. Large regions centered on the proximal and distal breakpoints (dashed lines) of the *XR* inversion show discordant clustering of *D*. *persimilis SR* with *D*. *pseudoobscura* rather than *D*. *persimilis ST*. *(B)* Large regions of phylogenetic discordance are not observed in the remainder of the genome.

Repetitive elements, such as *Leviathan* sequences, are known to be hotspots for inversion breakpoints [33,34]. While *Leviathan* repeats are unique to *D*. *persimilis* and *D*. *pseudoobscura*, *XR* alone harbors more than 650 of these repeats spread across the chromosome arm. Given this large number, the probability of a second inversion event (e.g. [35–37]) on *D*. *persimilis SR* at the same two *Leviathan* repeats as the original breakpoints appears vanishingly small. To directly test whether *D*. *persimilis SR* is recently derived from *D*. *persimilis ST* through a secondary inversion event, we inferred phylogenetic relationships in 10kb non-overlapping windows across the chromosome, using *D*. *miranda* as an outgroup. As expected, *D*. *persimilis SR* sequences cluster with those from *D*. *persimilis ST* across nearly the entire genome (Fig 2A). Surprisingly, we find two large blocks of phylogenetic discordance concentrated at the inversion breakpoints on *XR*. In these regions of phylogenetic discordance that span a few megabases of sequences, *D*. *persimilis SR* sequences are more closely related to *D*. *pseudoobscura* rather than to *D*. *persimilis ST*, with several regions within the inversion also showing the same discordant pattern (Fig 2B).

**Fig 3:**
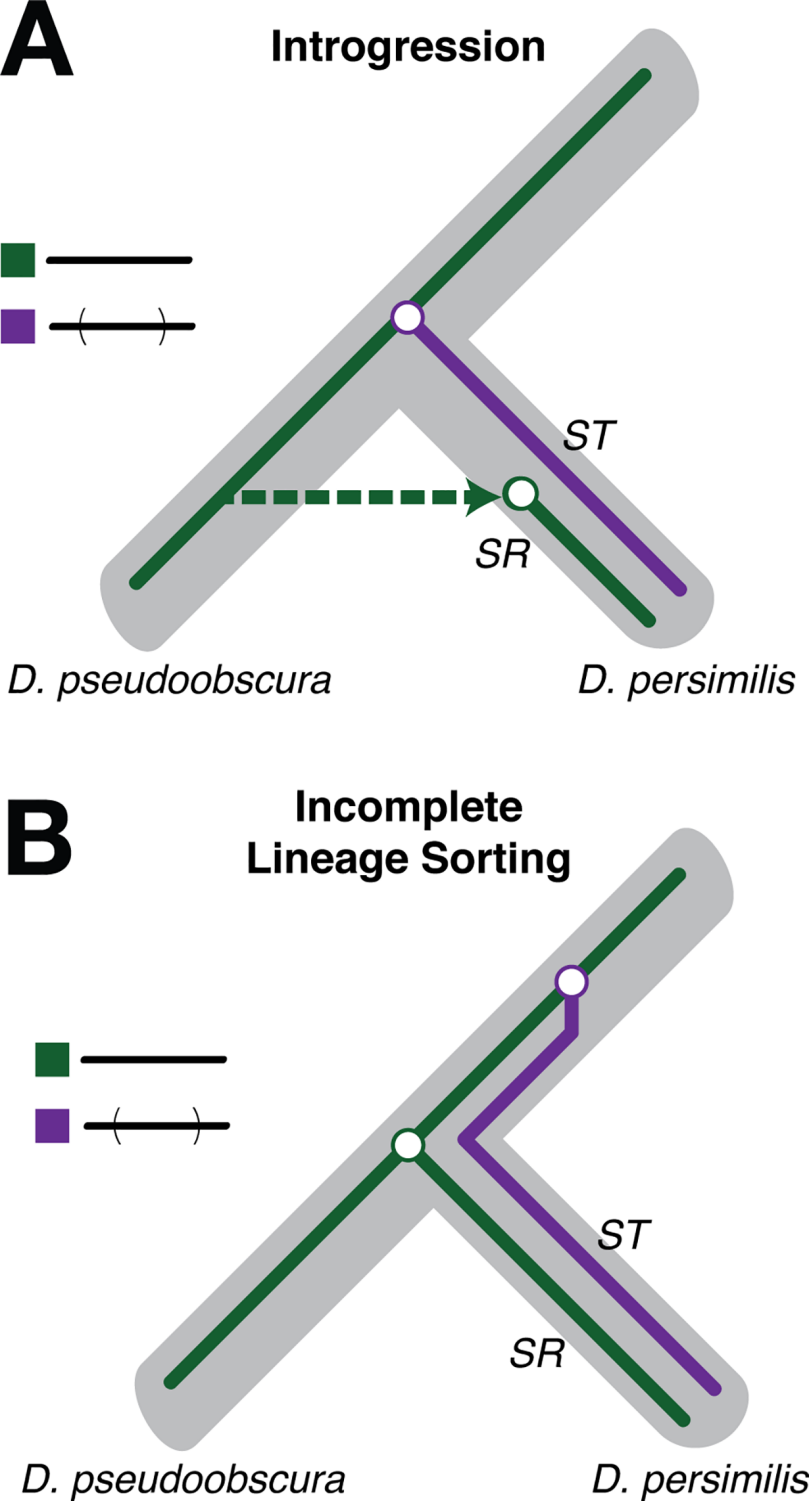
Discordance may be produced by introgression or incomplete lineage sorting of the *XR* arrangements. Under model *(A)*, the *D*. *persimilis ST* inversion segregates in the ancestral population of the species. Later divergence between *D*. *persimilis SR* and *D*. *pseudoobscura* chromosomes and recombination restriction between the two *D*. *persimilis* chromosomes leads to phylogenetic discordance at the inversion breakpoints. *(B)* An introgression model again predicts discordance if the *D*. *persimilis SR* chromosome introgressed from *D*. *pseudoobscura* after species divergence. Recombination between the introgressed chromosome *and D*. *persimilis ST* will gradually homogenize the two chromosomes excluding the inversion breakpoints.

We next asked whether the phylogenetic discordance observed on the *D*. *persimilis SR* chromosome is found anywhere else in the genome. Our sliding window phylogenetic analyses based on the *XR* classification (DpseST, DperST, and DperSR) show that there are no other large blocks of phylogenetic discordance anywhere else in the genome **(**Fig 2B). Although these analyses revealed small regions of phylogenetic discordance in other regions of the genome, there is no clustering of consecutive discordant windows, and the discordant windows are not associated with other fixed inversions. We also separately analyzed the *Standard* arrangement on the *3*^*rd*^ chromosome (*3*^*ST*^) which, like *D*. *persimilis SR*, is both shared across *D*. *persimilis* and *D*. *pseudoobscura* and is polymorphic within each species, and the *Arrowhead* arrangement (*3*^*AR*^) which is unique to *D*. *pseudoobscura*. Sequences at the breakpoints of this shared polymorphic inversion recapitulate the correct species tree, again indicating that the large blocks of phylogenetic discordance at the inversions breakpoints on *XR* are a unique property of the *D*. *persimilis SR* chromosome (S1 Text; S3 Fig). Together with the precisely-shared breakpoints, the relatedness between *D*. *persimilis SR* and *D*. *pseudoobscura* at the inversion breakpoints rejects the secondary-inversion hypothesis for the origin of the *D*. *persimilis SR* arrangement, and suggests a single origin for these chromosomes. Our results raise the surprising possibilities that *D*. *persimilis SR* was derived either through a recent introgression event from *D*. *pseudoobscura*, or from incomplete lineage sorting of the polymorphism from the common ancestor of *D*. *persimilis* and *D*. *pseudoobscura* (Fig 3).

### Regions of phylogenetic discordance allow a dating of free gene exchange between the *D*. *persimilis SR* and *D*. *pseudoobscura ST* arrangements

Because *D*. *persimilis* and *D*. *pseudoobscura* can potentially hybridize in nature [17], our results raise the possibility that the *D*. *persimilis SR* arrangement originated as a recent introgression of *D*. *pseudoobscura XR* (Fig 3A). Under the introgression scenario, repeated back-crossing to *D*. *persimilis* after the initial hybridization event gradually removes *D*. *pseudoobscura* material through single crossovers outside the inversion, and through double crossovers or gene conversion events inside the inversion. These recombination events homogenize *D*. *persimilis SR* and *ST*, largely wiping out any hints of a potential cross-species origin of *D*. *persimilis SR* from *D*. *pseudoobscura*. However, this history of introgression would be best preserved at the breakpoints of the inversion where suppression of crossovers is greatest [38,39]. The preservation of *D*. *pseudoobscura* material at the inversion breakpoints would then generate the blocks of phylogenetic discordance that we observe on *D*. *persimilis SR*.

An alternative explanation involving the inheritance of the *D*. *persimilis SR* and *D*. *pseudoobscura ST* arrangements from the common ancestor of both species is also consistent with the observed patterns. In particular, the phylogenetic discordance that we observe can be explained by the inheritance of the *D*. *persimilis SR* arrangement from the ancestor of *D*. *persimilis* and *D*. *pseudoobscura*, in combination with the loss of one arrangement from *D*. *pseudoobscura* (Fig 3B). Under this scenario of incomplete lineage sorting (ILS) in *D*. *persimilis*, the *ST* inversion originates as a segregating polymorphic chromosome in the ancestral population of *D*. *persimilis* and *D*. *pseudoobscura*. The recombination-suppressed regions at the breakpoints of the *D*. *persimilis ST* inversion begin diverging from the ancestrally arranged chromosomes long before the initial evolution of reproductive isolation. During this time, the ancestor of *D*. *persimilis SR* and *D*. *pseudoobscura ST* chromosomes (which are collinear) continue to freely recombine until the splitting of the two species, but diverge from the ancestor of the *D*. *persimilis ST* chromosome. Similar to the introgression scenario, recombination events homogenize the central regions of the *D*. *persimilis SR* and *ST* arrangements after speciation, except at the breakpoints of the inversion, thus leading to the patterns of phylogenetic discordance.

Common approaches to distinguish introgression from ILS, such as *f*-statistics and related “ABBA-BABA” methods, involve an implicit assumption of free recombination in the ancestral population. However, in the case of inversions and other recombination limited regions of the genome this assumption is violated and these measures cannot reliably distinguish between the two hypotheses. Alternatively, we can discriminate between these scenarios by determining whether the exchange occurred after the spitting of the two species (introgression) or in the ancestor of both species before the evolution of reproductive isolation (ILS). To estimate the date of exchange relative to reproductive isolation, we first estimated absolute divergence (*d*_*xy*_) in 10 kb windows for different regions of the genome. We then normalized *d*_*xy*_ in each window relative to the divergence with the *D*. *miranda* outgroup, a measure known as the “relative node depth” (RND), to adjust for regional variation in the mutation rate [40]. It is important to note that accurately converting absolute divergence to units of years is known to be fraught with several sources of error and requires an accurate calibration point in the absence of an estimate of the mutation rate in each species [41]. For the sake of interpretability, we scale the genetic differentiation in each window to the widely used *D*. *pseudoobscura*-*D*. *miranda* divergence time of 2 million years [42]. However, we rely on the relative comparison between distributions of *d*_*xy*_ and RND which are sufficient to resolve the questions we seek to address here.

*D*. *persimilis* and *D*. *pseudoobscura* are thought to have diverged approximately 500,000 years ago [15,42]. Indeed, in our data the average RND between *D*. *persimilis* and *D*. *pseudoobscura* in all collinear regions across the genome is 0.528 (95% CI: 0.521–0.535; Median: 0.513) and the mean divergence time based on genetic differentiation is estimated as 452,806 years ago (95% CI: 445,713–459,890). To determine the timing of chromosome exchange of the *D*. *persimilis SR/D*. *pseudoobscura ST* arrangements, we used the sequences flanking the inversion breakpoints (± 250 kb) to estimate divergence between *D*. *persimilis SR* and *D*. *pseudoobscura* and observe a mean RND of 0.662 (95% CI: 0.639–0.685; Median: 0.659). In these regions, we estimate the *D*. *persimilis SR* chromosome to have shared a common ancestor with *D*. *pseudoobscura ST* ~1 million years ago (95% CI: 0.95–1.05 Mya; Table 1). The estimated distribution of RND in these flanking regions is significantly greater (*P*<2.2x10^-16^, Wilcoxon rank-sum test) than the distribution of RND in collinear regions of the genome. Because the free exchange of the *D*. *persimilis SR/ D*. *pseudoobscura* ST arrangement appears to have occurred long before the time of species divergence, these results argue against a recent introgression event, and are consistent with incomplete lineage sorting of an ancestral chromosomal arrangement of the *D*. *persimilis SR*/ *D*. *pseudoobscura ST* arrangement in the ancestor of both species.

**Table 1:** Estimates of the relative ages of chromosomal inversions in *D*. *persimilis* and *D*. *pseudoobscura* relative to species divergence time. The fixed inversions on the *XL* and *2nd* chromosomes, as well as the polymorphic inversions on *XR* and the Pikes Peak (*3*^*PP*^) inversion arose before species divergence.

	*Species divergence*	*DperSR divergence*	*Chr XR inversion*	*Chr 2 inversion*	*Chr XL inversion*	*Chr 3 PP inversion*	*Chr 3 AR inversion*	*Chr 3 AR divergence*
Divergence time (mya)	0.45	1.00	1.14	1.55	1.65	0.75	0.42	0.59
Time prior to species divergence	0	0.55	0.69	1.10	1.20	0.30	-0.03	0.14

The inference that the *D*. *persimilis SR* and *D*. *pseudoobscura ST* chromosomes were freely segregating before the evolution of reproductive isolation between the two species suffers from two potential caveats. First, although some reproductive isolating mechanisms such as hybrid male sterility can potentially evolve quickly, speciation may be considered as a gradual process. Under this scenario, an estimate for the range of time rather than a point estimate for the evolution of reproductive isolation between *D*. *persimilis* and *D*. *pseudoobscura* may be more appropriate. Second, recent gene flow between the two species may lead to some degree of homogenization of the two genomes and a reduction in genomic divergence between the two species. This scenario may lead to an underestimate of the species divergence time. Nonetheless, in the absence of information regarding the genes that contribute to reproductive isolation between the species, there is little guidance for the degree to which the genomic divergence estimates must be adjusted to take into account gene flow after the evolution of reproductive isolation.

We, therefore, pursued a second independent line of enquiry that does not depend on inferences from sequence divergence or differentiation to test whether the *D*. *persimilis SR*/ *D*. *pseudoobscura ST* chromosomes freely segregated in the ancestor of both species before the evolution of reproductive isolation. Hybrid F1 males between *D*. *persimilis* and *D*. *pseudoobscura* are sterile in both directions of the cross, whereas all hybrid females are fully fertile. We determined whether the current day *D*. *pseudoobscura ST* can be transferred to *D*. *persimilis* through introgression to yield fertile hybrid males. We used marker assisted backcrossing to transfer the *D*. *pseudoobscura ST* chromosome into an otherwise *D*. *persimilis* genetic background. If these hybrid males are fertile, then this may provide strong evidence that introgression of the *D*. *pseudoobscura ST* arrangement into *D*. *persimilis* is potentially possible. Despite backcrossing for 15 generations and repeated testing of the fertility of the males from these crosses, all resulting hybrid males were sterile (S4 Fig). Consistent with previous studies, these results indicate the presence of strong hybrid male sterility genes on *D*. *pseudoobscura XR* [9,43–45]. These results further contradict the recent introgression scenario, and indicate that hybrid male sterility loci on *XR* must have evolved after these chromosomes were exchanged in the ancestor of both species. Together with the divergence estimates, these results are consistent with the idea that *D*. *persimilis SR* and *D*. *pseudoobscura* may have freely segregated in the ancestor of both species prior to the evolution of reproductive isolating loci on *XR*. More importantly, these results also allow us to provide a range estimate for speciation with a lower bound of approximately 450,000 years based on allelic divergence estimates in collinear regions, and an upper bound of approximately 1 million years ago.

### All fixed inversions in *D*. *persimilis* originated as segregating polymorphisms in the ancestral population of *D*. *persimilis* and *D*. *pseudoobscura*

**Fig 4:**
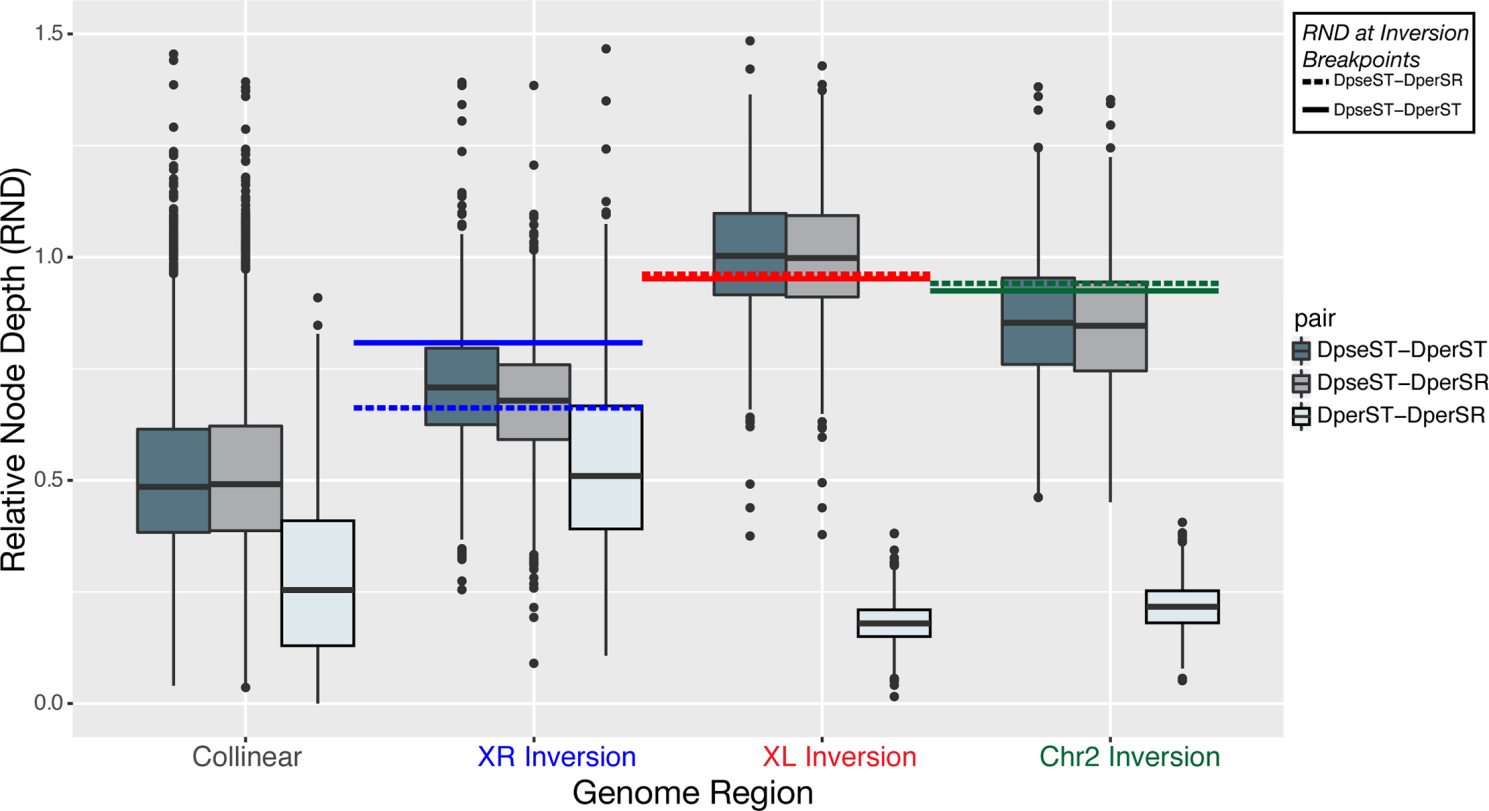
The distribution of divergence estimated across genomic regions. Divergence was estimated in 10 kb windows as the Relative Node Depth (RND; *d*_*xy*_ normalized to the outgroup) across the genome. The boxplots show the distribution of RND for each comparison in all collinear regions, and across the *XR*, *XL* and *2*^*nd*^ chromosome inversions. The horizontal lines depicted in the three fixed inversions indicate the mean RND estimated in the regions flanking the inversion breakpoints (±250 kb) for *D*. *pseudoobscura*-*D*. *persimilis* ST (solid) and *D*. *pseudoobscura*-*D*. *persimilis* SR (dashed).

**Fig 5:**
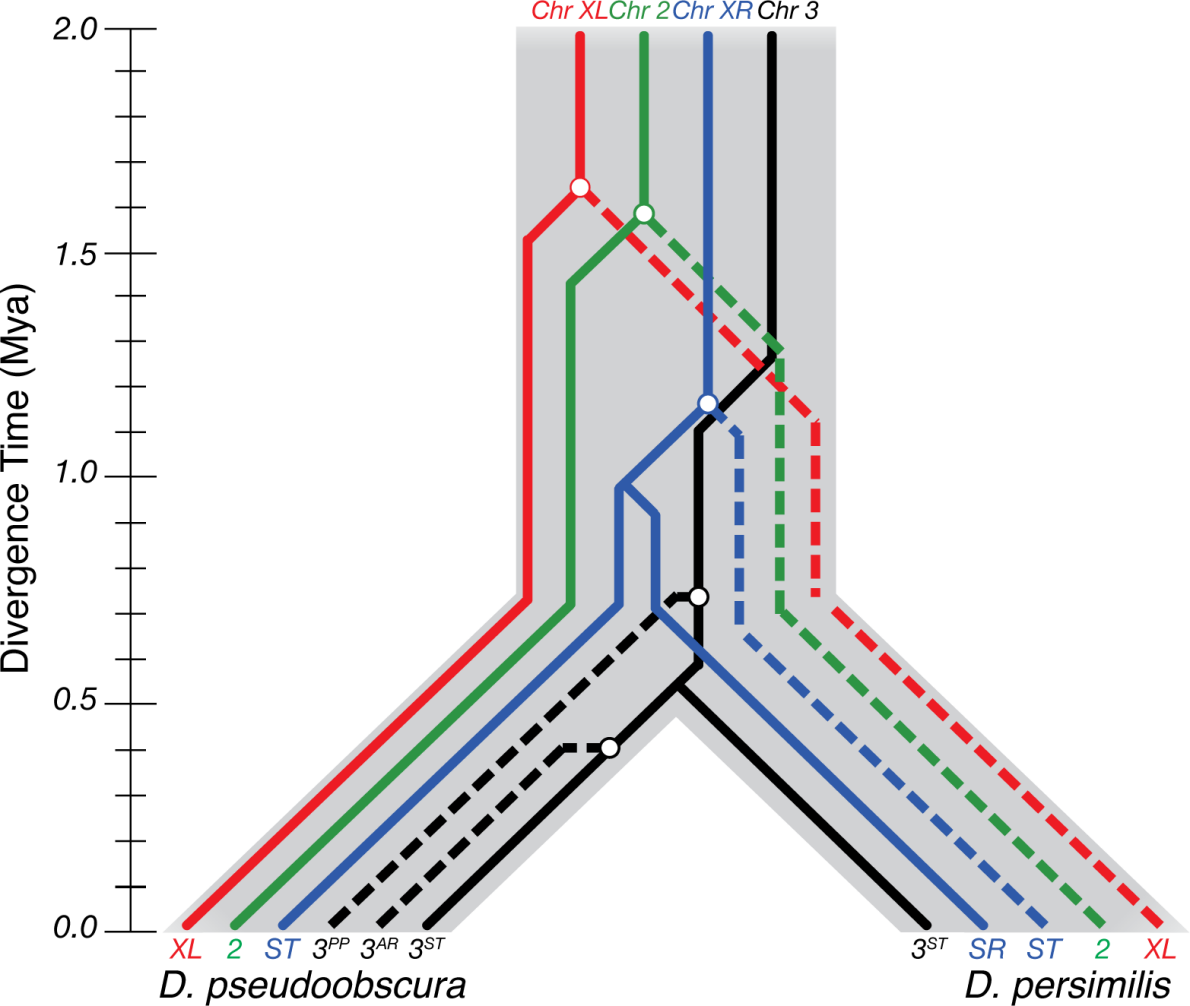
Incomplete lineage sorting of the inversions of *D*. *persimilis* and *D*. *pseudoobscura*. The fixed inversions on the *XL* and *2nd* chromosomes, as well as the polymorphic inversions on *XR* and the Pikes Peak (*3*^*PP*^) inversion arose before species divergence. Incomplete lineage sorting produced the observed inversion patterns in the species present today.

Because the *XR* inversion polymorphism exists only in *D*. *persimilis* and not in *D*. *pseudoobscura*, it is often assumed that this inversion must have originated in the *D*. *persimilis* lineage after the splitting of the two species [31,46]. The idea that the *XR* inversion on the *Standard* chromosome of *D*. *persimilis* originated as a segregating polymorphic inversion in the ancestral population prior to speciation goes against what is widely-accepted, although this scenario has been hypothesized previously [11]. The two other fixed inversions on the *XL* and *2*^*nd*^ chromosomes in *D*. *persimilis* are thought to be even older than the *XR* inversion [11,46,47]. We estimated divergence between *D*. *pseudoobscura* and *D*. *persimilis ST* in sequences flanking the *XL* and *2*^*nd*^ chromosome inversion breakpoints, and, consistent with previous studies [11,46,47], observed greater levels of divergence for both fixed inversions (RND_*XL*_: 0.962, 95% CI: 0.941–0.983; RND_*2*_:0.941, 95% CI:0.923–0.959) than for *XR* (RND_*XR*_: 0.808, 95% CI: 0.776–0.840) as the distribution of RND was significantly increased for each (*P<*2.2x10^-16^,Wilcoxon rank-sum test; Fig 4). A similar pattern is observed for the median levels of RND in each inversion (RND_*XL*_: 0.958; RND_*2*_:0.937, RND_*XR*_: 0.780). The median *D*. *pseudoobscura—D*. *persimilis ST* RND for each inversion is more than double the genome-wide median RND (RND_*Genome*_: 0.259). Likewise, scaling genetic differentiation to the speciation time with *D*. *miranda*, we estimate that the inversions on *XL* and the *2*^*nd*^ chromosomes originated approximately 1.64 ± 0.41 and 1.55 ± 0.24 million years ago, respectively (Table 1; Fig 5). From the analysis of *D*. *pseudoobscura* and *D*. *persimilis ST* divergence in 10kb sliding windows, we observe a significant overrepresentation of RND estimates in the top 1% genome-wide across all three inversions relative to collinear regions (*χ*^2^ = 208.3, *P*<2x10^-16^; S5 Fig). The proportion of RND windows in the top 1% is greatest on the *XL* inversion, followed by the *2*^*nd*^ chromosome inversion, with the fewest across the *XR* inversion (S5 Fig). Our results suggest that all of these fixed inversions originated in the ancestral population before the speciation event that separated *D*. *persimilis* and *D*. *pseudoobscura*. Furthermore, the relative divergence and differentiation pattern of *XL* > 2 > *XR* that we infer is consistent with findings from previous studies [10,47].

The difference in divergence and differentiation of the fixed inversions and collinear regions is not subtle (Fig 4): the *XL*, *XR* and *2*^*nd*^ chromosome inversions are nearly twice as old as the estimates for collinear regions between the two species and the distributions of RND are significantly greater for each (Fig 4, S5 Fig). The increased divergence we observe in the fixed *XL*, *XR*, and *2*^*nd*^ inversions is not a novel finding and has been well documented by others [11,46,47]. Although the possibility of these inversions arising in the ancestral population has previously been raised, all studies to date have concluded that the reduced divergence in collinear regions is the result of gene flow upon secondary contact and that all inversions must have originated after speciation [11,12,14,15,46,48]. To test if the fixed inversions originated as segregating polymorphisms in the ancestral species as our results suggest, we modeled divergence and gene flow under alternative evolutionary scenarios of speciation.

Using loci sampled from intergenic regions across inverted and collinear regions of the genome, we fit our data to models of strict divergence in isolation, isolation-with-migration (IM), and isolation-with-initial-migration (IIM) with maximum-likelihood estimation [49]. In collinear regions the IIM model gave a significantly better fit than the IM model or a null model of strict divergence (Table 2), providing further evidence of post-speciation gene flow as supported by several previous studies [11,12,14,15,46,48]. Under the IIM model, the estimated time of population divergence in inverted regions should represent its origin [50]. To test if the inversions are associated with an older population divergence time than collinear regions and therefore predate the species split, we allowed the parameters of the IIM model to vary independently between each inversion and collinear regions and compared the results to a fully constrained model where the parameters are fixed between regions [50]. The model allowing for individual parameters to differ between regions fit the data significantly better (*χ*^2^ = 26.2, *P*<8.6x10^-6^), indicating that the *XL*, *XR* and *2*^*nd*^ inversions arose prior to the population divergence in collinear regions and further supporting the idea that they existed as ancestral polymorphisms (S6 Fig). For each inversion, the parameter estimate for the population divergence time *t*_*0*_ is greater than in collinear regions, although we note the confidence intervals overlap for the case of *XR*. However, we find evidence to support that *t*_*0*_ is different between the *XR* inversion and collinear regions, as a model where we allow parameters to vary in each fits the data significantly better than a constrained model where divergence parameters are held constant (2Δ*lnL* = -6.76;*P<*3.4x10^-2^). In each region, we estimate one-way gene flow from *D*. *pseudoobscura* to *D*. *persimilis* and no migration in the other direction (S6 Fig). Although we find evidence for gene flow from *D*. *pseudoobscura* to *D*. *persimilis* after speciation in agreement with several previous studies [11,12,14,15,46,48], we do not conclude this is solely responsible for the pattern of increased divergence observed across fixed inversion differences. Instead, these results indicate that all of the fixed, derived inversions in *D*. *persimilis* must have freely segregated in the ancestral population for a substantial period of time before the reproductive barriers were complete.

**Table 2:** Maximum likelihood support and likelihood ratio tests for gene flow under models of speciation. The log-likelihoods are displayed for isolation (Iso), isolation-with-migration (IM), and isolation-with-initial-migration (IIM) models. The estimates in bold correspond to the maximum likelihoods for each genomic region. In each case, the IIM model has the best support. The columns labeled Iso and IM show the likelihood ratio test statistics for each model relative to the IIM model.

	*lnL*_*Iso*_	*lnL*_*IM*_	*lnL*_*IIM*_	*Iso*	*IM*
Full Genome	-19000.18	-18899.42	-18698.87	77.81	51.79
Collinear	-13848.79	-13689.17	-13591.07	66.55	25.34
Chr XL Inversion	-1390.10	-1390.10	-1372.71	4.49	4.49
Chr 2 Inversion	-1429.95	-1429.95	-1408.24	18.52	18.52
Chr XR Inversion	-2251.10	-2241.11	-2225.54	6.61	4.02

## Discussion

The study of chromosomal inversions in the classic systems of *D*. *pseudoobscura* and *D*. *persimilis* has deeply informed our understanding of the evolutionary forces that shape natural variation, the evolution of new species, and selfish chromosome dynamics. Our results have important implications for all of these fields. We provide a resolution to the strange collinearity of the *D*. *persimilis SR* and *D*. *pseudoobscura ST* chromosomes first observed by Dobzhansky [24,51]. We show that this collinearity is a consequence of the direct descent of these chromosomes from one of the ancestrally segregating arrangements, and not due to two independent inversions at the same breakpoints. Our results also provide evidence that pervasive gene flow after the initial evolution of reproductive isolation is not necessarily required to explain the observed phylogenetic discordance. A similar maintenance of chromosomal arrangements across species resulting from an ancient inversion polymorphism has also been demonstrated in *Anopheles* mosquitos [52]. Segregation distorters are often associated with inversions because new inversions that tightly link a segregation distorter gene with existing enhancer alleles enjoy a selective advantage [27]. In contrast to most other *Sex-Ratio* systems associated with derived inversions, our results suggest that the *D*. *persimilis SR* system evolved on the background of an ancestral arrangement. Similarly, recent studies of the *t*-haplotype in *M*. *musculus* also support an ancient origin of inversions associated with segregation distortion [53]. These results indicate that segregation distorters may not only become associated with new inversions, as is traditionally thought, but can also arise on the genetic backgrounds of existing chromosome inversion polymorphisms.

In addition to clarifying the evolutionary history of *Sex-Ratio* chromosome in *D*. *persimilis*, the age estimates of the fixed chromosomal inversion differences in *D*. *pseudoobscura* and *D*. *persimilis* suggest a new role of chromosomal inversions in the evolution of reproductive isolation genes. Any model exploring this role must explain at least two empirical patterns: a) the fixed inversions between *D*. *persimilis* and *D*. *pseudoobscura* have higher divergence as compared to collinear regions of the genome, and b) most genes that underlie reproductive isolation between *D*. *persimilis* and *D*. *pseudoobscura* reside within these inversion differences [7–9]. Previous work in this species pair reconciled these empirical observations with a model where inversions arise after speciation and secondary contact between taxa homogenizes collinear regions [11]. Thus, previous models explained the role of chromosomal inversions in speciation as protectors of hybrid incompatibly alleles from the homogenizing force of extensive hybridization [9,11]. Instead, we show that these inversions were freely segregating in the ancestral population long before the complete isolation of *D*. *pseudoobscura* and *D*. *persimilis*, and that genes contributing to reproductive barriers must have evolved within them afterwards.

**Fig 6:**
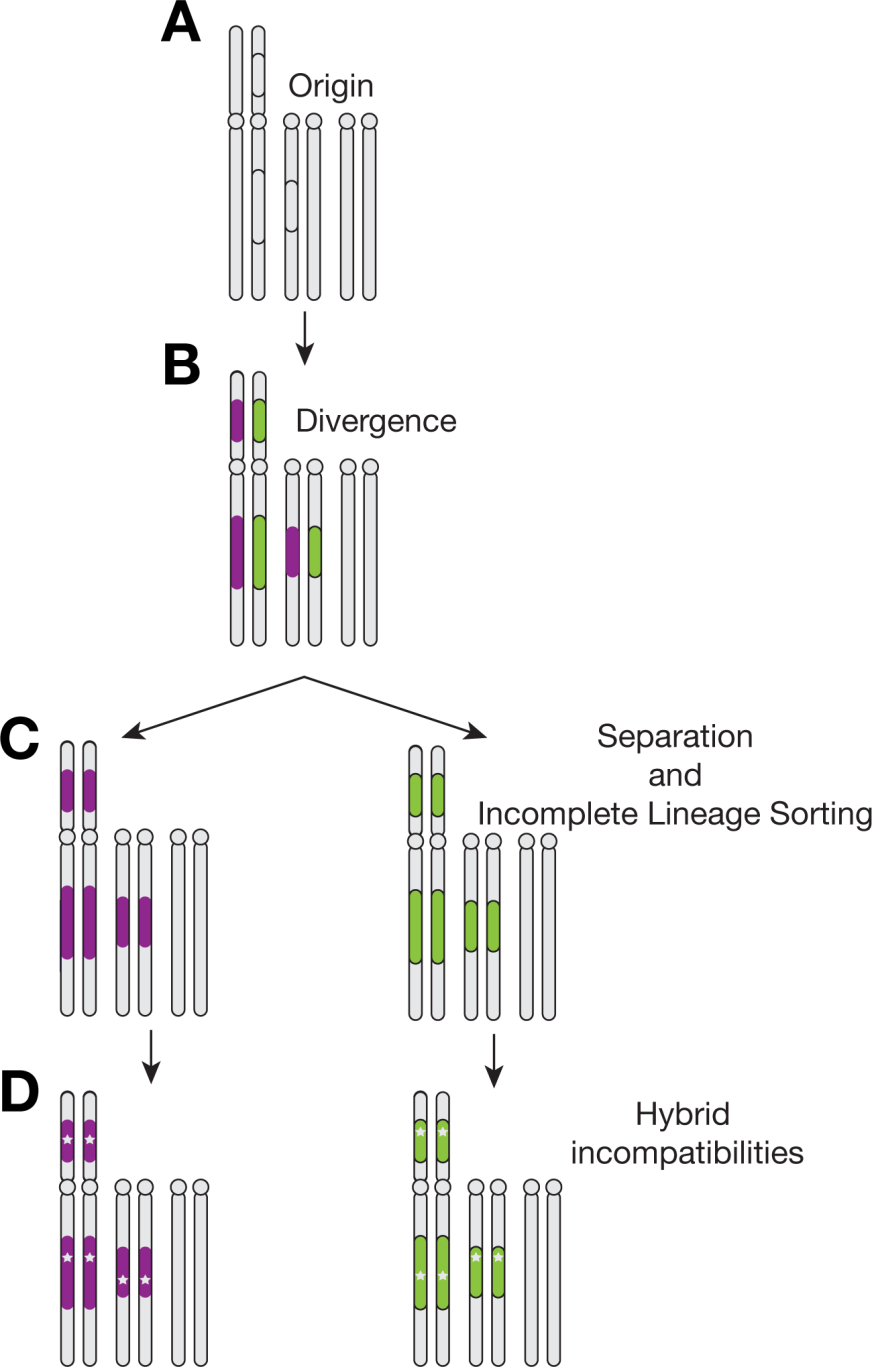
Inversions accelerate the formation of hybrid incompatibilities. *(A)* Polymorphic inversions arise in the ancestor of the two species. *(B)* Restricted recombination between the inversions leads to accumulating divergence (red, blue) distinct from collinear regions of the genome (grey). *(C)* Incomplete sorting of the inversions between two isolated populations generates immediate divergence between the two populations. *(D)* Preexisting divergence increases the chance of hybrid incompatibilities forming in the inverted regions as compared to the collinear regions.

Here, we propose a simple model under which ancestrally segregating inversions that undergo incomplete lineage sorting can lead to high allelic divergence at these inversions, which may in turn accelerate the formation of hybrid incompatibilities (Fig 6). Chromosomal inversions can arise and persist in ancestral populations [18]. During this period, the genomic regions spanning the inversions and the corresponding regions on the un-inverted chromosomes can accumulate genetic divergence aided by the suppression of recombination in heterozygotes [18,54–57]. Populations with ancient segregating inversions diverge within inverted regions, but stay genetically similar in collinear regions [54,57]. These chromosomal inversions may undergo incomplete lineage sorting if the ancestral population is split into two allopatric populations [58]. At the initial time of separation, all loci across collinear and inverted backgrounds start as equally compatible, the genes in collinear regions are nearly identical, while genes within the chromosomal inversions are already highly diverged. This accumulation of hybrid incompatibilities occurs in isolation, unopposed by the selective cost of producing unfit offspring, and in a manner consistent with the Dobzhansky-Muller model [59,60]. The collinear regions will retain their low divergence signature from the ancestral population until speciation is complete. Under this model, the heterogeneity in divergence across the genome caused by ancestrally segregating inversions makes the evolution of alleles that cause reproductive isolation more likely in the regions encompassed by these inversions rather than in the collinear regions of the genome.

Our reasoning that highly diverged genes may evolve to an incompatible state more quickly than those with little divergence rests on the implicit assumption that the evolution of hybrid incompatibilities requires multiple genetic changes. This view, although somewhat speculative, is supported by three lines of evidence. First, theory shows that changes at a minimum of two genes are required to produce a hybrid incompatibility, and that it may be easier to evolve more complex incompatibilities that involve changes at multiple genes [61]. These ideas have strong empirical support [1]. For example, the genetic architecture of hybrid sterility between *D*. *pseudoobscura pseudoobscura* and *D*. *pseudoobscura bogotana*–one of the youngest hybridizations to be studied–involves a single hybrid incompatible interaction between *at least* six genes [45]. Second, nearly all hybrid incompatibility genes that have been identified so far show the rapid accumulation of many amino acid changes, and represent some of the most highly diverged genes in the genome [62,63]. Ultra-fine scale mapping studies that dissect how many of these changes within these genes contribute to hybrid sterility or hybrid inviability have not yet been performed. However, there are no known cases of hybrid incompatibility genes that involve one or only a few amino acid changes. Third, both theory and empirical data show that hybrid incompatibilities accumulate faster than linearly with divergence between populations [64–66]. Populations that display higher genomic divergence are, therefore, more likely to have evolved hybrid incompatibilities as compared to those that have little or no genomic divergence [67]. Together, these lines of evidence support the idea that the evolution of hybrid incompatibilities is a multi-step process. By accumulating genetic divergence even before the initial population split, the genes associated with ancestrally segregating chromosomal inversions may be fewer steps away from reaching an incompatible state. In contrast, genes in collinear regions of the genome show little or no divergence between recently split populations and must start accumulating changes from scratch if they are to eventually an incompatible state.

The idea that chromosomal inversions are often associated with hybrid incompatibility genes is a widely-held view among evolutionary geneticists [18,68]. There are four lines of evidence for the widespread acceptance of this association. First, direct genetic mapping of loci that underlie reproductive barriers may show these genes to be located in genomic regions that harbor fixed chromosomal inversions [9,43,69,70]. Such genetic studies provide the most direct line of evidence for a potential association of reproductive isolation genes with chromosomal inversions. Second, genomic regions spanning chromosomal inversions often show signatures of higher divergence or reduced introgression [11,19,46]. As our results show, this line of evidence may be susceptible to erroneous interpretations when the evolutionary histories and the ages of these inversions are unknown. Third, sympatric species show higher incidence of fixed inversions than allopatric species. While there are limited data supporting such a pattern [9,47,71,72], this line of evidence for the association of hybrid incompatibility genes with chromosomal inversions is indirect and prone to observational biases. Fourth, theoretical studies show that it may be possible for hybrid incompatibility genes to evolve and persist despite gene flow during or after speciation [20,73]. These theoretical results, however, are not a good substitute for direct empirical evidence. We, therefore, consider direct genetic mapping studies that localize reproductive isolation genes to regions spanning chromosomal inversions as the most reliable line of evidence supporting the association of chromosomal inversions with reproductive isolation genes. Such genetic studies that map loci that contribute to reproductive isolating barriers, and overlay those loci on the locations of chromosomal inversions are surprisingly rare. To our knowledge, the only direct study of this nature in animal taxa involves the *D*. *pseudoobscura-D*. *persimilis* hybridization, where genetic mapping studies have shown that loci that contribute to reproductive isolation are enriched, but not exclusively located, on chromosomes that also carry fixed inversion differences between these species [9,47,60,74,75]. In the absence of other such studies, it is not clear whether this pattern is specific to this particular species pair, or is a broadly held pattern. We, therefore, find that the amount of evidence for the association of hybrid incompatibility genes with fixed chromosomal inversions is not proportionate to how widely this pattern is believed to be true.

This paucity of genetic mapping studies to determine the locations of hybrid incompatibility genes relative to chromosomal inversions is not entirely surprising. A necessary step in understanding the molecular basis of speciation involves the identification of the genes that contribute to reproductive barriers. Most speciation geneticists who aim to identify such genes may either focus on studying species pairs that lack chromosomal inversion differences, or abandon such studies when these genes map to chromosomal inversions because there is little hope of precisely identifying the causal genes. Fortunately, uncovering evidence for an association of reproductive isolation genes with chromosomal inversions requires neither the precise identification of the genes nor determining the precise breakpoints of chromosomal inversions. Coarse mapping of quantitative trait loci that underlie reproductive isolation across several species pairs, and overlaying these loci with the approximate locations of chromosomal inversion differences between these species may prove sufficient to establish the generality of this pattern [69].

In summary, we propose that incomplete lineage sorting of ancestrally segregating polymorphisms can establish patterns of higher divergence within chromosomal inversions, and may potentially promote the evolution of hybrid incompatibilities in these highly diverged regions. Our model can explain previously observed empirical patterns even in cases where there is no evidence for gene flow across populations during or after speciation. Together, these ideas force a reconsideration of the role of chromosomal inversions in speciation, perhaps not as protectors of existing hybrid incompatibility alleles, but as fertile grounds for their formation.

## Materials and methods

### Isolation and maintenance of Sex-Ratio chromosome strains

Wild caught *D*. *persimilis* strains were provided as a generous gift by Dean Castillo, collected in the Sierra Nevada mountain range and near Mt. St. Helena, CA. We tested individuals from these strains for the presence of *Sex-Ratio* chromosomes by crossing males to standard *D*. *persimilis* females. We isolated two individual *D*. *persimilis Sex-Ratio* strains and generated stable stocks through eight to twelve generations of inbreeding. All stocks were raised on standard cornmeal media at 18 degrees C.

### Polytene chromosome analyses

We used two crosses of *D*. *persimilis SR*/*ST* heterozygotes to compare the *D*. *persimilis SR* chromosome with *D*. *pseudoobscura* and *D*. *persimilis ST* chromosomes. In the first cross, a *D*. *persimilis SR*/*ST sepia* (*se*) heterozygous female was crossed to a *D*. *pseudoobscura ST se* male. Of the two *XL*/*XR* karyotypes possible from this cross, we examined females heterozygous for *XL* and homozygous for *XR* inversions. These females allow us to evaluate whether the *D*. *persimilis SR* and *D*. *pseudoobscura ST* chromosomes are homosequential. In a second cross, a *D*. *persimilis SR*/*ST se* heterozygous female was crossed to a *D*. *persimilis ST se* male. Of the two *XL*/*XR* karyotypes possible from this cross, we examined females homozygous for *XL* and heterozygous for *XR* inversions. These females allow us to examine the *D*. *persimilis SR* and *D*. *persimilis ST* heterozygotes. We prepared salivary squashes from larvae from these two crosses using standard techniques, with modifications described by Harshman (1977) and Ballard and Bedo (1991) [76–78].

### DNA extraction and sequencing

To generate whole genome shotgun sequencing libraries for *D*. *persimilis* strains, we pooled one male each from two *SR* strains and two *ST* strains (from Sierra Nevada and Mt St Helena collections). We extracted DNA from these flies using the 5 Prime Archive Pure DNA extraction kit according to the manufacturer’s protocol (ThermoFisher, Waltham, MA). All libraries were generated with the Illumina TruSeq Nano kit (Epicentre, Illumina Inc, CA) using the manufacturers protocol, and sequenced as 500bp paired end reads on an Illumina HiSeq 2000 instrument.

### Sequence alignment and SNP identification

Low-quality bases were removed from the ends of the raw paired end reads contained in FASTQ files using *seqtk* (https://github.com/lh3/seqtk) with an error threshold of 0.05. Illumina adapter sequences and polyA tails were trimmed from the reads using Trimmomatic version 0.30 [79]. The read quality was then manually inspected using FastQC. Following initial preprocessing and quality control, the reads from each pool were aligned to the *D*. *pseudoobscura* reference genome (v 3.2) using *bwa* version 0.7.8 with default parameters [80]. Genome wide, the average fold coverage was ~180x and ~133x for the *D*. *persimilis ST* and *SR* pools, respectively (S1 Table). For reads mapping to X chromosome scaffolds, the average fold coverage was ~97x and ~74x for *D*. *persimilis ST* and *SR*, respectively (S2 Table).

After the binary alignments were sorted and indexed with SAMtools [81], single nucleotide polymorphisms (SNPs) were called using *freebayes* (v. 0.9.21; [82] with the expected pairwise nucleotide diversity parameter set to 0.01, based on a previous genome-wide estimate from *D*. *pseudoobscura* [55]. The samples were modeled as discrete genotypes across pools by using the “–J” option and the ploidy was set separately for *X* chromosome scaffolds (1*N*) and autosomes (2*N*). SNPs with a genotype quality score less than 30 were filtered from the dataset. We restricted all downstream analyses to sites that had coverage greater than *1N* and less than 3 standard deviations away from the genome wide mean for all samples (S1 Table). Across the genome we identified a total of 3,598,524 polymorphic sites, 703,908 and 844,043 of which were located on chromosomes *XR* and *XL*, respectively.

The *D*. *pseudoobscura* reference assembly does not contain complete sequences for either of the arms of the *X* or *4*^*th*^ chromosomes. Instead, each is composed of a series of scaffold groups that differ both in size and orientation relative to one another [83]. Schaeffer et al. (2008) previously determined the approximate locations and ordering of each of these scaffolds [83]. We used their map to convert the scaffold-specific coordinates of each site to the appropriate location on the corresponding chromosome to construct a continuous sequence.

### Estimating the phylogenetic relationship of Sex-Ratio chromosomes

We estimated the genetic distance between each pairwise grouping in 10 kb windows using Nei’s *D*_*A*_ distance, which has been shown to accurately recover the topology of phylogenetic trees from allele frequency data [84,85]. To root the tree with an outgroup, we aligned publically available short reads of *D*. *miranda* (SRX965461; strain SP138) to the *D*. *pseudoobscura* reference genome. In each window, we constructed neighbor-joining trees [86] using distance matrices constructed from the estimated genetic distances (*D*_*A*_) and classified the phylogeny based on the topology it supported. If a window contained fewer than 10 segregating sites, we did not construct a tree or estimate the genetic distance. For each tree we performed 10,000 bootstrap replicates and only included those windows with a support value of 0.75 or higher.

### Divergence estimates

We estimated absolute allelic divergence with Nei’s *d*_*xy*_, a measure of the average number of pairwise nucleotide substitutions per site [87,88]. *d*_*xy*_ was measured between each population grouping in 10 Kb, nonoverlapping windows across the genome. Each comparison was then normalized to the divergence with the outgroup *D*. *miranda* in the same window to account for regional mutational differences, a measure known as the “relative node depth” [40]. Confidence intervals were determined from 1000 bootstrap replicates of windows in each region under consideration. Divergence time estimates were obtained with the Cavalli-Sforza transformation of *F*_*ST*_ as

$$T=-log(1-F_{ST})$$

and then multiplied by a scaling factor in each window so that the divergence time between *D*. *pseudoobscura* and *D*. *miranda* was equal to 2 Mya [42,89–91].

### Modeling gene flow

To test for evidence of post-speciation gene flow we considered three different models: (i) strict divergence in isolation (Iso) with an instantaneous split of an ancestral population at time *t*_*0*_ without any gene flow after, (ii) isolation-with-migration (IM) where an ancestral population split into two subpopulations at time *t*_0_ with constant migration rates *M*_*1*_ and *M*_*2*_ between them afterwards, and (iii) isolation-with-initial-migration (IIM) where gene flow is restricted to occur over a time *V* after the initial split, ceasing at time *t*_*1*_. We used the methods derived by Costa and Wilkinson-Herbots (2017) to obtain maximum-likelihood estimates for the parameters under each model. Becquet and Przeworski (2009) and Strasburg and Rieseberg (2010), among others, have shown that parameter estimation with IM models can be unreliable if assumptions concerning population structure and recombination are broken [92,93]. While the maximum-likelihood method of Costa and Wilkinson-Herbots has been demonstrated to be robust to demographic misspecification, we nonetheless do not rely on this analysis to provide accurate parameter estimates of divergence times and instead use the approach to test for the relative support among speciation models. Some previous studies have suggested that gene flow between *D*. *pseudoobscura* and *D*. *persimilis* has occurred upon secondary contact more recently after initial isolation, however the IIM model has been shown to approximate the dynamics of this scenario reasonably well [49]. To remove potential confounding effects of selection, we restricted our analysis to intergenic noncoding regions of each chromosome. We then randomly sampled 500 bp segments that were separated by a minimum of at least 10 kb to create a set of loci for each region, similar to the multilocus dataset of Wang and Hey (2010). The coalescent models of Costa and Wilkinson-Herbots (2017) require separate estimates of pairwise differences in loci (i) within *D*. *pseudoobscura*, (ii) within *D*. *persimilis*, and (iii) between *D*. *pseudoobscura* and *D*. *persimilis*. Therefore, we randomly divided the loci for each analysis into three nonoverlapping datasets. Relative mutation rates are also required for each locus. Here, as recommended by Costa and Wilkinson-Herbots (2017), we used the divergence (i.e. *d*_*xy*_) to the outgroup *D*. *miranda* to estimate these relative mutation rates [94,95].

We used likelihood ratio tests to determine the relative support for each model, where the difference in log-likelihood between models 2Δ*lnL* is assumed to follow a *χ*^2^ distribution with the number of degrees of freedom equal to the difference between the dimensions of parameter space of the two models. The maximum-likelihood estimates for each model can be computed rapidly because linkage is assumed to be negligible between loci. Thus, to correct for the statistical effect of LD between loci, we scaled the difference in *lnL* between models by a factor of 1/*x* as in Lohse et al. (2015), where *x* is the average number of loci sampled in each 100 kb region (*x* = 7.75). To test if the fixed inversions are older than the species split we allowed individual parameters of the IIM model to vary between collinear regions, and each of the *XL*, *XR* and *2*^*nd*^ inversions. We then compared this complex model to a constrained model, where each parameter was fixed across the genome, similar to the hierarchical model testing in Lohse et al. (2015). The confidence intervals reported for each parameter are the Wald confidence intervals computed from the inverted Hessian matrix of the maximum-likelihood estimators [49].

### Identification and verification of inversion breakpoints

The proximal and distal breakpoints have both been characterized previously, and the regions in *D*. *pseudoobscura* contain unique sequence flanking a series of 302-bp repeats known as Leviathan repeats, present throughout the genomes of both *D*. *pseudoobscura* and *D*. *persimilis*. We designed primers to capture both the array of repeats as well as portions of unique sequence. We extracted DNA from all three genotypes and amplified the proximal breakpoint region using primers designed to anneal to the *D*. *pseudoobscura* genomic sequence flanking the Leviathan repeats (F5’- GATCTAATCCAGAAAGTTCGCTTGCG -3’, R5’- AGTGTGACCCATTTTAAGCGG-3’). These primers amplified a single, approximately 1500bp, product in *D*. *pseudoobscura* and *D*. *persimilis SR*, but not *D*. *persimilis ST*. PCR products were Sanger sequenced using the forward and reverse PCR primers at the DNA Sequencing Core Facility, University of Utah. The reads were aligned both to one another and to sequence from the *D*. *pseudoobscura* genome assembly around the proximal breakpoint. The sequenced PCR product was confirmed to contain both the repeats and sections of the unique sequence flanking the repeat region at the proximal breakpoint.

## Supporting information

S1 Text Supplementary methods for phylogenetic and divergence analyses in *D*. *pseudoobscura*. This text details the methods used to analyze phylogenetic discordance on the third chromosome of *D*. *pseudoosbcura* and *D*. *persimilis*. Further, this text contains the methods used to determine the relative age of the *Arrowhead* (*3*^*AR*^) and *Pikes Peak* (*3*^*PP*^*)* arrangements in *D*. *pseudoobscura*.
(PDF)

S1 Fig Polytene squash of a *D*. *persimilis ST/SR* female heterozygote. The *XR* chromosome is contains a single inversion as observed by a characteristic inversion loop. The remainder of the genome is homosequential.
(PDF)

S2 Fig PCR amplification of the proximal breakpoint. Genomic template from *D*. *pseudoobscura* and *D*. *persimilis SR*, but not *D*. *persimilis ST*, generated an approximately 1.5kb amplicon of the proximal breakpoint with primers specific for the ancestral orientation of the *XR* chromosome.
(PDF)

S3 Fig Species clustering within inversion polymorphisms on chromosome *3*. The *D*. *pseudoobscura* 3^rd^ chromosome arrangements Standard (*ST*) and Arrowhead (*AR*) lack the large breakpoint-specific phylogenetic discordance observed at the inversion break points of the inversion between *D*. *pseudoobscura* and *D*. *persimilis SR* on chromosome *XR*. While some windows demonstrate phylogenetic discordance, these windows are independent of the arrangement of the chromosome forms and, unlike the *XR* inversion, do not cluster at the inversion breakpoints.
(PDF)

S4 Fig Introgression of the D. *pseudoobscura ST* arrangement into a *D*. *persimilis* genetic background. Despite 15 generations of marker-assisted backcrossing, all hybrid males that carry the *D*. *pseudoobscura XR* material in an otherwise *D*. *persimilis* genetic background are sterile. These results indicate that the chromosome-level gene exchange must have happened before the evolution of hybrid incompatibilities on this chromosome arm.
(PDF)

S5 Fig Divergence in sliding windows across chromosomes. Smoothing splines are shown for divergence measured as relative node depth (RND) in 10kb windows across chromosomes XR (A), XL (B), and 2 (C). The different colors for each line indicate the taxa pair RND is estimated for, with the key in the legend. Colored dots represent individual windows that are in the top 1% of RND values genome-wide and are considered outliers. Black vertical lines indicate the locations of inversion breakpoints on each chromosome. The insets on XR show a close-up view of RND estimated around the proximal and distal inversion breakpoints ± 250 kb.
(PDF)

S6 Fig Isolation with initial migration model. The width of the bars are proportional to the population sizes and the heights of bars indicate time using the maximum likelihood approach of Costa and Wilkinson-Herbots (2017). The ancestral population for each set of data is indicated by a single colored bar that splits into two subpopulations at time t_0_. From t_0_ to t_1_ (V) the populations diverge in allopatry with the estimated levels of gene flow (M; in units of number of migrants per generation). At time t_1_, the populations no longer exchange genes among the subpopulations. The vertical white bars are the confidence intervals for time t_0_ and t_1_. The collinear region represents species divergence, while XR, 2, and XL represent the divergence of fixed inversion differences between *D*. *pseudoobscura* and *D*. *persimilis*.
(PDF)

S1 Table *D*. *pseudoobscura* and *D*. *persimilis* reference alignment statistics. Statistics are presented for the total number of reads mapped to the *D*. *pseudoosbcura* reference genome for each sample and the *D*. *miranda* outgroup.
(PDF)

S2 Table Reference alignment statistics for each sample across scaffolds. Each scaffold in the *D*. *pseudoobscura* reference genome is listed, with corresponding coverage and mapping statistics for each sample and the *D*. *miranda* outgroup.
(PDF)
