# Supplementary material for: Ancestral polymorphisms explain the role of chromosomal inversions in speciation
Source: PLoS Genet. 2018 Jul 30;14(7):e1007526. doi: 10.1371/journal.pgen.1007526 (PMC6085072; doi:10.1371/journal.pgen.1007526)
Supplement: S1 Text — This text details the methods used to analyze phylogenetic discordance on the third chromosome of D. pseudoosbcura and D. persimilis. Further, this text contains the methods used to determine the relative age of the Arrowhead (3AR) and Pikes Peak (3PP) arrangements in D. pseudoobscura. (PDF) [file pgen.1007526.s001.pdf]

S1 Text

Supplemental Text for:

The role of chromosomal inversions in speciation

Fuller Z.L.[1,2*], Leonard, C.J.[3*], Young, R.E.[3, 4], Schaeffer, S.W.[1], & Phadnis, N[3**].

* these authors contributed equally

[1] Department of Biology, 208 Erwin W. Mueller Laboratories, The Pennsylvania State University, University Park, PA 16802.

[2] Current address: 606 Fairchild Center, Department of Biological Sciences, Columbia University, New York, NY 10027

[3] Department of Biology, University of Utah, Salt Lake City, UT 84112.

[4] Current address: Department of Genetics, University of Wisconsin, Madison, WI 53706.

** address correspondence to:
Nitin Phadnis, 257 South 1400 East, Department of Biology, University of Utah, Salt Lake City, UT 84112; tel: (801)585-0493 email: nitin.phadnis@utah.edu

SUPPLEMENTARY METHODS

Phylogenetic Analysis Using Polymorphic Third Chromosome Inversions of D. pseudoobscura

In both species the third chromosome is highly polymorphic for a series of inversions, including one that is shared between them, referred to as the "standard" arrangement [1,2]. The wild-caught samples included in our study carry unknown third chromosome arrangements, however the reference strains of *D. pseudoobscura* and *D. persimilis* carry the "arrowhead" (AR) and standard arrangements respectively [3]. In order to test if the phylogenetic pattern and significant evidence of introgression observed surrounding the SR breakpoints are simply an artifact of polymorphic inversions that are shared among species, we performed a similar analysis using the *D. persimilis* reference sequence and known *D. pseudoobscura* arrangements.

To date, the *D. persimilis* reference strain MSH-3 has not been resequenced and the original reads used for the genome assembly available from the NCBI Trace Archive were generated from Sanger sequencing. As a result, reads from MSH-3 could not be aligned to the *D. pseudoobscura* reference sequence using the same bioinformatics pipeline as the samples included in our study. Instead, we aligned *D. pseudoobscura* reads to the *D. persimilis* reference genome and called SNPs with the same method used to generate variants in our original analysis. For the *D. pseudoobscura* sequences, we obtained reads (Accession Number: PRJNA358242) of 14 lines carrying the AR arrangement and 8 lines carrying the standard arrangement that were each made homozygous for the third chromosome. Similar to the X chromosome and chromosome 4 of the *D. pseudoobscura* reference assembly, the reference of chromosome 3 for *D. persimilis* is distributed among 9 large scaffolds. Using the map determined by Schaeffer et al. (2008), we converted the scaffold specific coordinates to their appropriate location on the third chromosome to construct a continuous sequence. Furthermore, the location and coordinates of the breakpoints for the ST/AR inversion have previously been determined for the *D. persimilis* reference assembly (19). We then performed a similar test for discordant phylogenies in 10 kb windows across the third chromosome (Figure S3). Although a small number of discordant phylogenies are detected within the AR inversion, it does not appear there are significant excesses of any topology.

Estimates of the polymorphic 3rd chromosome inversions of D. pseudoobscura.

In *D. pseudoobscura*, the *Arrowhead* (3^{AR}) and *Pikes Peak* (3^{PP}) arrangements are both derived from 3ST, although 3^{AR} is estimated to be one of the younger arrangements and 3^{PP} is relatively old [5]. To confirm the relative timeline of the origin of these inversions and to clarify the evolutionary history of the arrangements in the ancestral population, we further estimated divergence between the *D. pseudoobscura* 3ST and derived polymorphic 3^{AR} and 3^{PP} 3rd chromosome inversions. Indeed, after standardizing the estimates of d_{xy} to the speciation time with *D. miranda,* we estimate that 3^{AR} is the youngest arrangement having diverged from *D. pseudoobscura* 3ST approximately 418 Kya. Furthermore, 3^{PP} appears to be older, diverging from 3ST ~750 Kya and before the species split between *D. pseudoosbcura* and *D. persimilis* (Table 1). These results suggest that 3^{PP} was segregating as a polymorphic inversion in the

ancestral species and was transmitted exclusively to *D. pseudoobscura* while the inversions on *XL* and the *2nd* chromosome were inherited by the *D. persimilis* lineage (Figure 4).

Supplemental References:

1. Schaeffer SW, Goetting-Minesky MP, Kovacevic M, Peoples JR, Graybill JL, Miller JM, et al. Evolutionary genomics of inversions in Drosophila pseudoobscura: Evidence for epistasis. Proc Natl Acad Sci U S A. 2003;100: 8319–8324. doi:10.1073/pnas.1432900100

2. Dobzhansky TG, Epling CC. Contributions to the Genetics, Taxonomy, and Ecology of Drosophila Pseudoobscura and Its Relatives. Th. Dobzhansky and Carl Epling. - Washington 1944. 8°. Carnegie Institution of Washington; 1944.

3. Richards S, Liu Y, Bettencourt BR, Hradecky P, Letovsky S, Nielsen R, et al. Comparative genome sequencing of Drosophila pseudoobscura: Chromosomal, gene, and cis-element evolution. Genome Res. 2005;15: 1–18. doi:10.1101/gr.3059305

4. Schaeffer SW, Bhutkar A, McAllister BF, Matsuda M, Matzkin LM, O'Grady PM, et al. Polytene Chromosomal Maps of 11 Drosophila Species: The Order of Genomic Scaffolds Inferred From Genetic and Physical Maps. Genetics. 2008;179: 1601–1655. doi:10.1534/genetics.107.086074

5. Wallace AG, Detweiler D, Schaeffer SW. Evolutionary History of the Third Chromosome Gene Arrangements of Drosophila pseudoobscura Inferred from Inversion Breakpoints. Mol Biol Evol. 2011;28: 2219–2229. doi:10.1093/molbev/msr039
