# Supplementary figures and images for: Ancestral polymorphisms explain the role of chromosomal inversions in speciation
Source: PLoS Genet. 2018 Jul 30;14(7):e1007526. doi: 10.1371/journal.pgen.1007526 (PMC6085072; doi:10.1371/journal.pgen.1007526)

DperSR/DperST

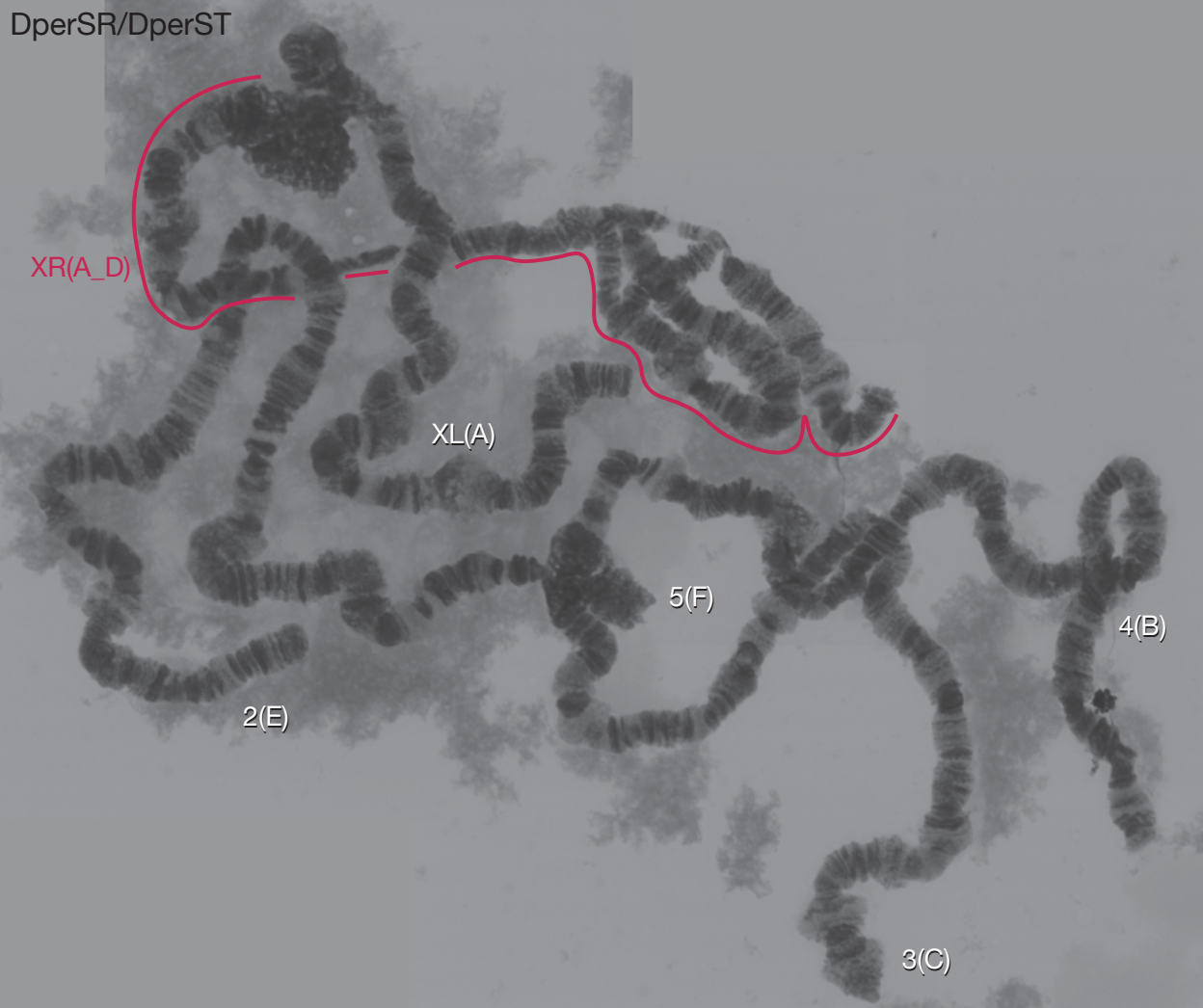

XR(A\_D)

XL(A)

2(E)

5(F)

4(B)

3(C)

Supplement: S1 Fig — The XR chromosome is contains a single inversion as observed by a characteristic inversion loop. The remainder of the genome is homosequential. (PDF) [file pgen.1007526.s002.pdf]

**A**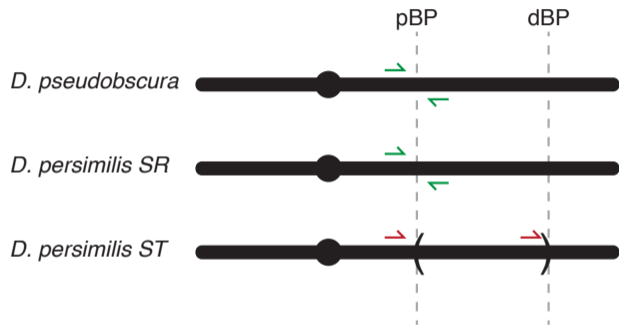**B**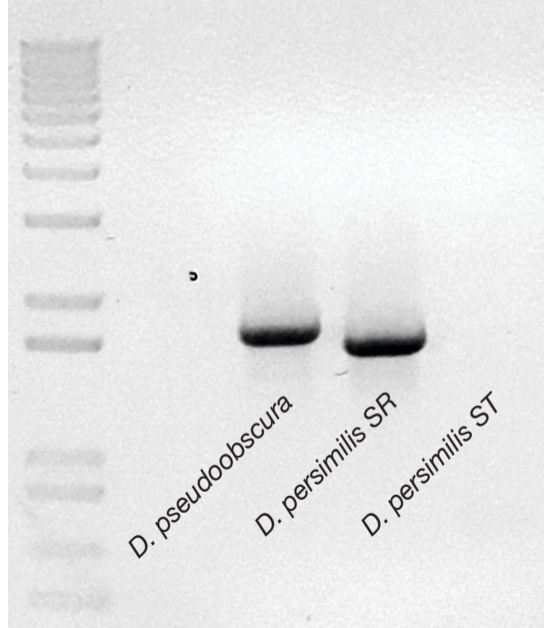

Supplement: S2 Fig — Genomic template from D. pseudoobscura and D. persimilis SR, but not D. persimilis ST, generated an approximately 1.5kb amplicon of the proximal breakpoint with primers specific for the ancestral orientation of the XR chromosome. (PDF) [file pgen.1007526.s003.pdf]

# Chromosome 3

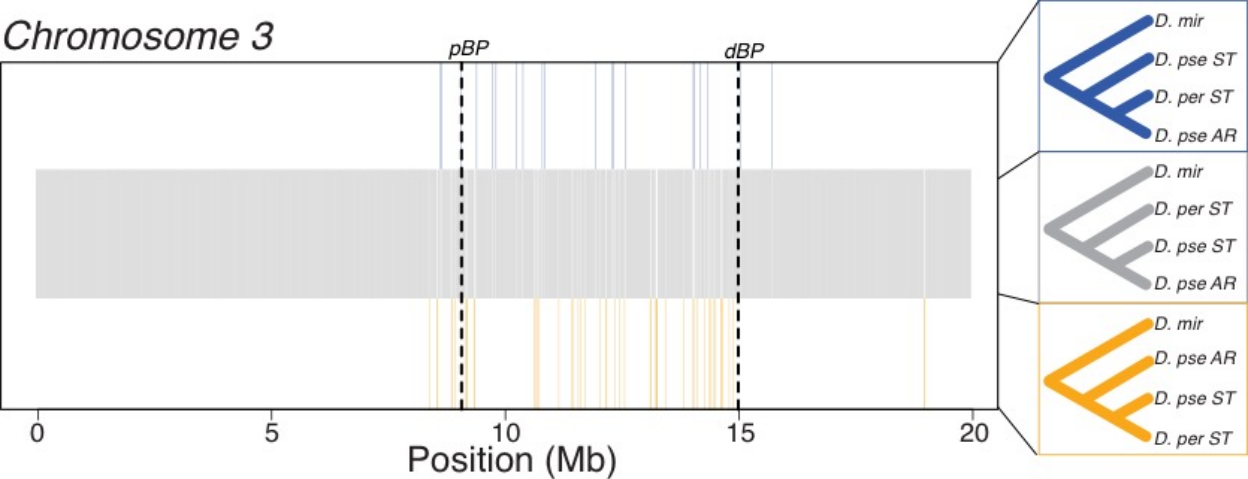

Supplement: S3 Fig — The D. pseudoobscura 3rd chromosome arrangements Standard (ST) and Arrowhead (AR) lack the large breakpoint-specific phylogenetic discordance observed at the inversion break points of the inversion between D. pseudoobscura and D. persimilis SR on chromosome XR. While some windows demonstrate phylogenetic discordance, these windows are independent of the arrangement of the chromosome forms and, unlike the XR inversion, do not cluster at the inversion breakpoints. (PDF) [file pgen.1007526.s004.pdf]

*D. persimilis* *se* females

*D. pseudoobscura* *se*<sup>+</sup> males

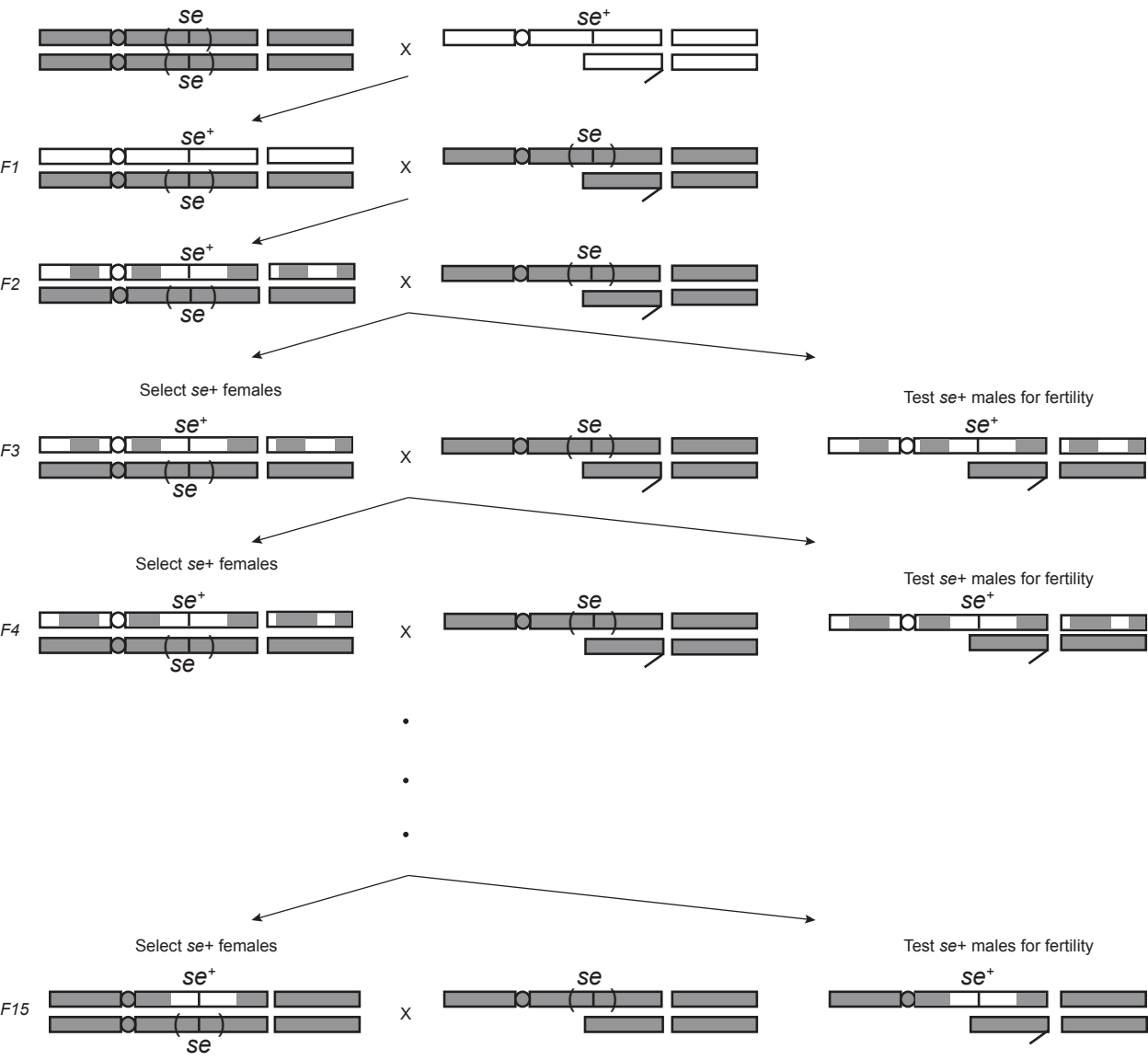

Supplement: S4 Fig — Despite 15 generations of marker-assisted backcrossing, all hybrid males that carry the D. pseudoobscura XR material in an otherwise D. persimilis genetic background are sterile. These results indicate that the chromosome-level gene exchange must have happened before the evolution of hybrid incompatibilities on this chromosome arm. (PDF) [file pgen.1007526.s005.pdf]

**A****Chromosome XR**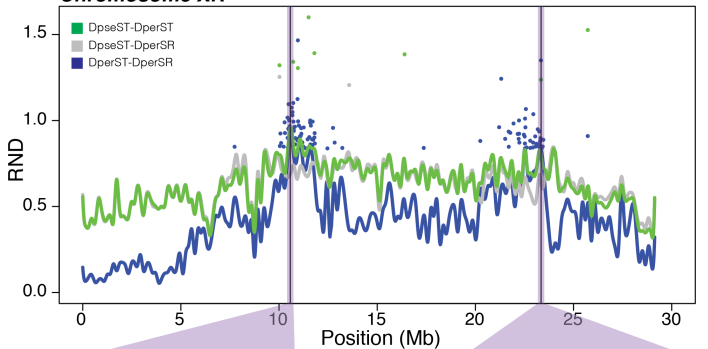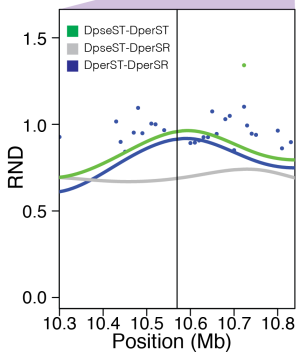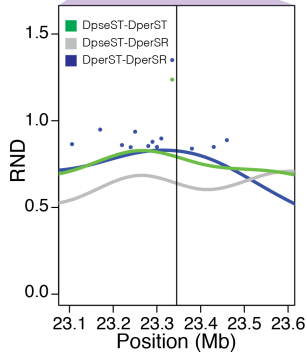**B****Chromosome XL**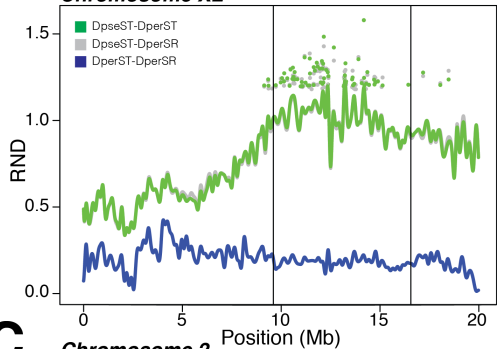**C****Chromosome 2**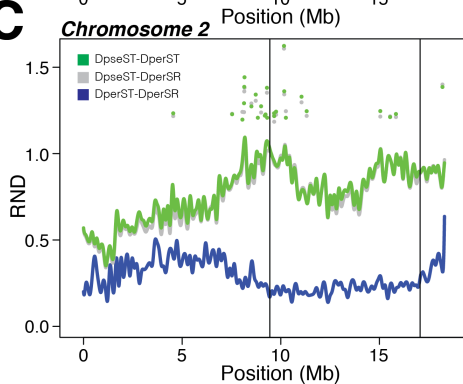

Supplement: S5 Fig — Smoothing splines are shown for divergence measured as relative node depth (RND) in 10kb windows across chromosomes XR (A), XL (B), and 2 (C). The different colors for each line indicate the taxa pair RND is estimated for, with the key in the legend. Colored dots represent individual windows that are in the top 1% of RND values genome-wide and are considered outliers. Black vertical lines indicate the locations of inversion breakpoints on each chromosome. The insets on XR show a close-up view of RND estimated around the proximal and distal inversion breakpoints ± 250 kb. (PDF) [file pgen.1007526.s006.pdf]

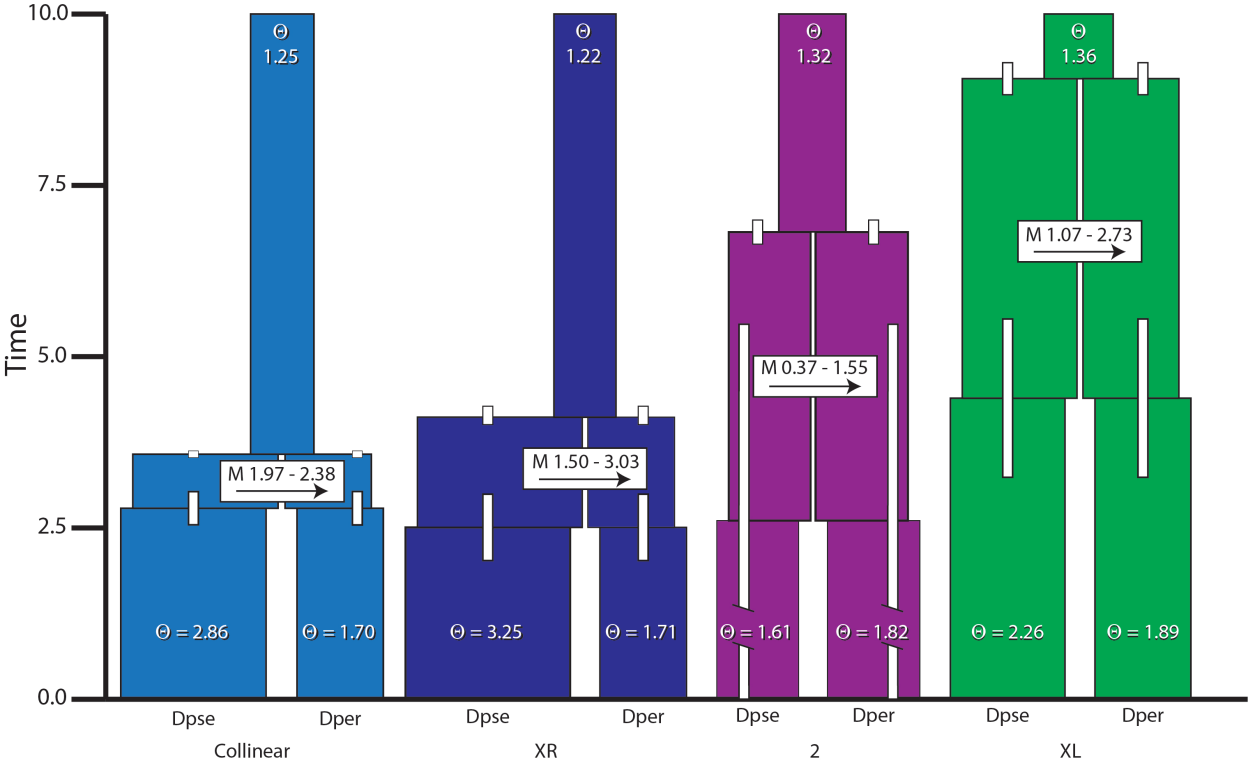

Supplement: S6 Fig — The width of the bars are proportional to the population sizes and the heights of bars indicate time using the maximum likelihood approach of Costa and Wilkinson-Herbots (2017). The ancestral population for each set of data is indicated by a single colored bar that splits into two subpopulations at time t0. From t0 to t1 (V) the populations diverge in allopatry with the estimated levels of gene flow (M; in units of number of migrants per generation). At time t1, the populations no longer exchange genes among the subpopulations. The vertical white bars are the confidence intervals for time t0 and t1. The collinear region represents species divergence, while XR, 2, and XL represent the divergence of fixed inversion differences between D. pseudoobscura and D. persimilis. (PDF) [file pgen.1007526.s007.pdf]
