# Supplementary material for: Ancestral polymorphisms explain the role of chromosomal inversions in speciation
Source: PLoS Genet. 2018 Jul 30;14(7):e1007526. doi: 10.1371/journal.pgen.1007526 (PMC6085072; doi:10.1371/journal.pgen.1007526)
Supplement: S1 Table — Statistics are presented for the total number of reads mapped to the D. pseudoosbcura reference genome for each sample and the D. miranda outgroup. (PDF) [file pgen.1007526.s008.pdf]

Supplemental Table 1: *D. pseudoosbcura* reference alignment statistics

Statistic	***D. pse ST***	***D. per ST***	***D. per SR***	***D. mir***
Total Reads	139337556	242743626	215111160	49649299
Mapped Reads	133512488	223244577	165073083	45556023
% Mapped	95.82	91.97	76.74	91.53
Mean Coverage	82.72	180.28	133.37	22.19
St. Dev. Coverage	236.03	666.46	538.60	60.15
% Reference Bases Covered (QUAL > 30, DP > 2)	96.49	95.48	95.45	89.77
