# Supplementary material for: Ancestral polymorphisms explain the role of chromosomal inversions in speciation
Source: PLoS Genet. 2018 Jul 30;14(7):e1007526. doi: 10.1371/journal.pgen.1007526 (PMC6085072; doi:10.1371/journal.pgen.1007526)
Supplement: S2 Table — Each scaffold in the D. pseudoobscura reference genome is listed, with corresponding coverage and mapping statistics for each sample and the D. miranda outgroup. (PDF) [file pgen.1007526.s009.pdf]

Supplemental Materials for:

The role of chromosomal inversions in speciation

Fuller Z.L.[1,2*], Leonard, C.J.[3*], Young, R.E.[3, 4], Schaeffer, S.W.[1], & Phadnis, N[3**].

* these authors contributed equally

[1] Department of Biology, 208 Erwin W. Mueller Laboratories, The Pennsylvania State University, University Park, PA 16802.

[2] Current address: 606 Fairchild Center, Department of Biological Sciences, Columbia University, New York, NY 10027

[3] Department of Biology, University of Utah, Salt Lake City, UT 84112.

[4] Current address: Department of Genetics, University of Wisconsin, Madison, WI 53706.

** address correspondence to:
Nitin Phadnis, 257 South 1400 East, Department of Biology, University of Utah, Salt Lake City, UT 84112; tel: (801)585-0493 email: nitin.phadnis@utah.edu

SUPPLEMENTARY METHODS

Phylogenetic Analysis Using Polymorphic Third Chromosome Inversions of D. pseudoobscura

In both species the third chromosome is highly polymorphic for a series of inversions, including one that is shared between them, referred to as the "standard" arrangement [1,2]. The wild-caught samples included in our study carry unknown third chromosome arrangements, however the reference strains of *D. pseudoobscura* and *D. persimilis* carry the "arrowhead" (AR) and standard arrangements respectively [3]. In order to test if the phylogenetic pattern and significant evidence of introgression observed surrounding the SR breakpoints are simply an artifact of polymorphic inversions that are shared among species, we performed a similar analysis using the *D. persimilis* reference sequence and known *D. pseudoobscura* arrangements.

To date, the *D. persimilis* reference strain MSH-3 has not been resequenced and the original reads used for the genome assembly available from the NCBI Trace Archive were generated from Sanger sequencing. As a result, reads from MSH-3 could not be aligned to the *D. pseudoobscura* reference sequence using the same bioinformatics pipeline as the samples included in our study. Instead, we aligned *D. pseudoobscura* reads to the *D. persimilis* reference genome and called SNPs with the same method used to generate variants in our original analysis. For the *D. pseudoobscura* sequences, we obtained reads (Accession Number: PRJNA358242) of 14 lines carrying the AR arrangement and 8 lines carrying the standard arrangement that were each made homozygous for the third chromosome. Similar to the X chromosome and chromosome 4 of the *D. pseudoobscura* reference assembly, the reference of chromosome 3 for *D. persimilis* is distributed among 9 large scaffolds. Using the map determined by Schaeffer et al. (2008), we converted the scaffold specific coordinates to their appropriate location on the third chromosome to construct a continuous sequence. Furthermore, the location and coordinates of the breakpoints for the ST/AR inversion have previously been determined for the *D. persimilis* reference assembly (19). We then performed a similar test for discordant phylogenies in 10 kb windows across the third chromosome (Figure S3). Although a small number of discordant phylogenies are detected within the AR inversion, it does not appear there are significant excesses of any topology.

Estimates of the polymorphic 3rd chromosome inversions of D. pseudoobscura.

In *D. pseudoobscura*, the *Arrowhead* (3^{AR}) and *Pikes Peak* (3^{PP}) arrangements are both derived from 3ST, although 3^{AR} is estimated to be one of the younger arrangements and 3^{PP} is relatively old [5]. To confirm the relative timeline of the origin of these inversions and to clarify the evolutionary history of the arrangements in the ancestral population, we further estimated divergence between the *D. pseudoobscura* 3ST and derived polymorphic 3^{AR} and 3^{PP} 3rd chromosome inversions. Indeed, after standardizing the estimates of d_{xy} to the speciation time with *D. miranda,* we estimate that 3^{AR} is the youngest arrangement having diverged from *D. pseudoobscura* 3ST approximately 418 Kya. Furthermore, 3^{PP} appears to be older, diverging from 3ST ~750 Kya and before the species split between *D. pseudoosbcura* and *D. persimilis* (Table 1). These results suggest that 3^{PP} was segregating as a polymorphic inversion in the

ancestral species and was transmitted exclusively to *D. pseudoobscura* while the inversions on *XL* and the *2nd* chromosome were inherited by the *D. persimilis* lineage (Figure 4).

SUPPLEMENTARY FIGURES

Supplemental Figure 1: **Polytene squash of a *D. persimilis ST/SR* female heterozygote.** The *XR* chromosome is contains a single inversion as observed by a characteristic inversion loop. The remainder of the genome is homosequential.

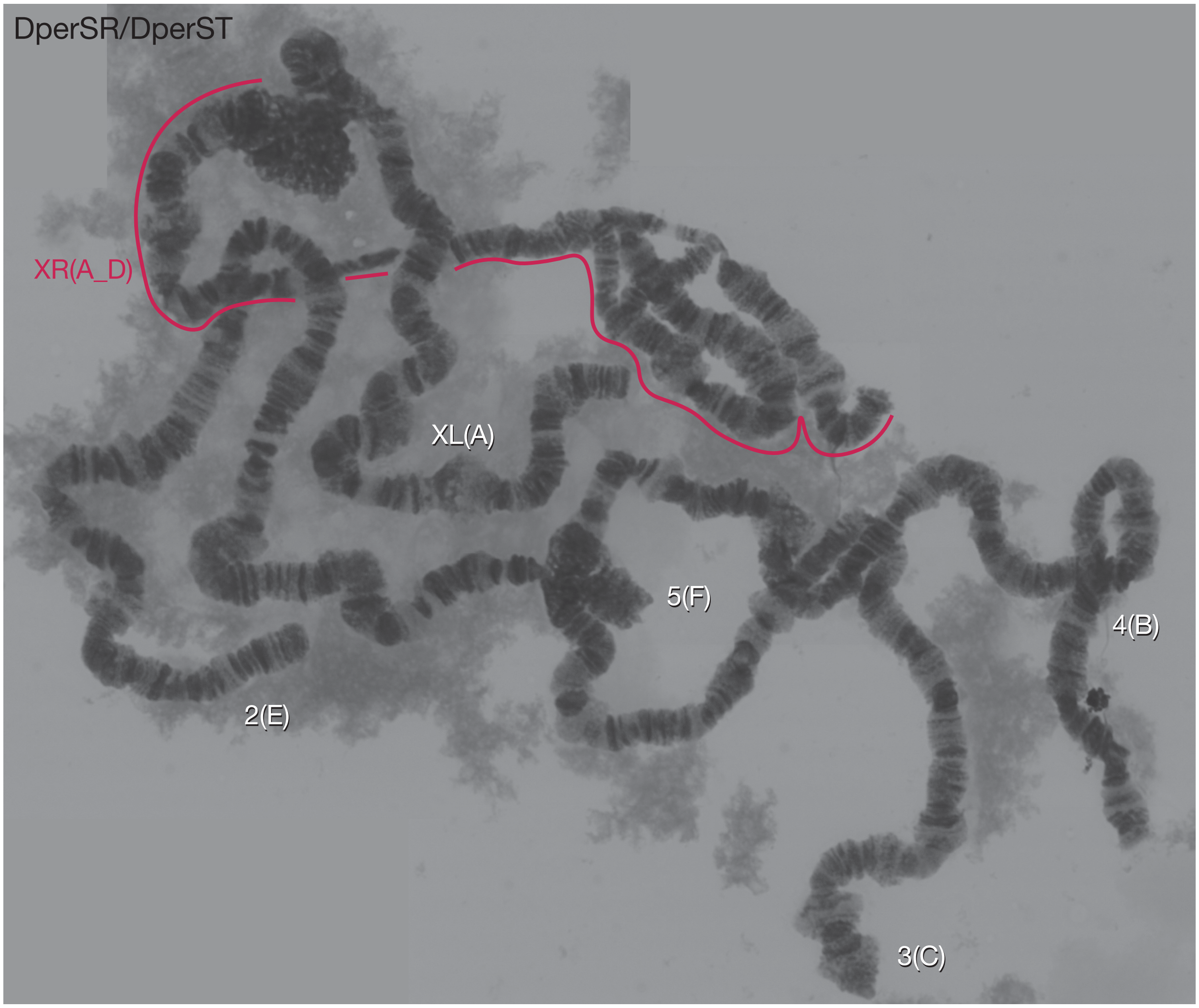

Supplemental Figure 2: **PCR amplification of the proximal breakpoint.** Genomic template from *D. pseudoobscura* and *D. persimilis SR*, but not *D. persimilis ST*, generated an approximately 1.5kb amplicon of the proximal breakpoint with primers specific for the ancestral orientation of the *XR* chromosome.

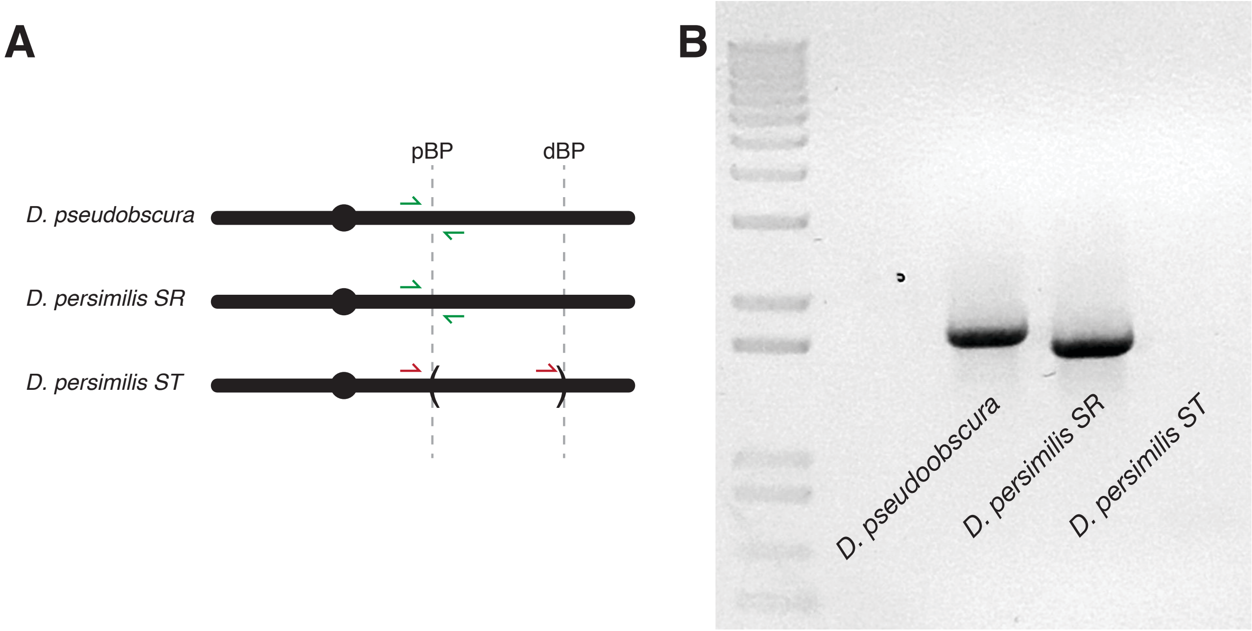

Supplemental Figure 3: **Species clustering within inversion polymorphisms on chromosome *3*.** The *D. pseudoobscura* 3rd chromosome arrangements Standard (*ST*) and Arrowhead (*AR*) lack the large breakpoint-specific phylogenetic discordance observed at the inversion break points of the inversion between *D. pseudoobscura* and *D. persimilis SR* on chromosome *XR*. While some windows demonstrate phylogenetic discordance, these windows are independent of the arrangement of the chromosome forms and, unlike the *XR* inversion, do not cluster at the inversion breakpoints.

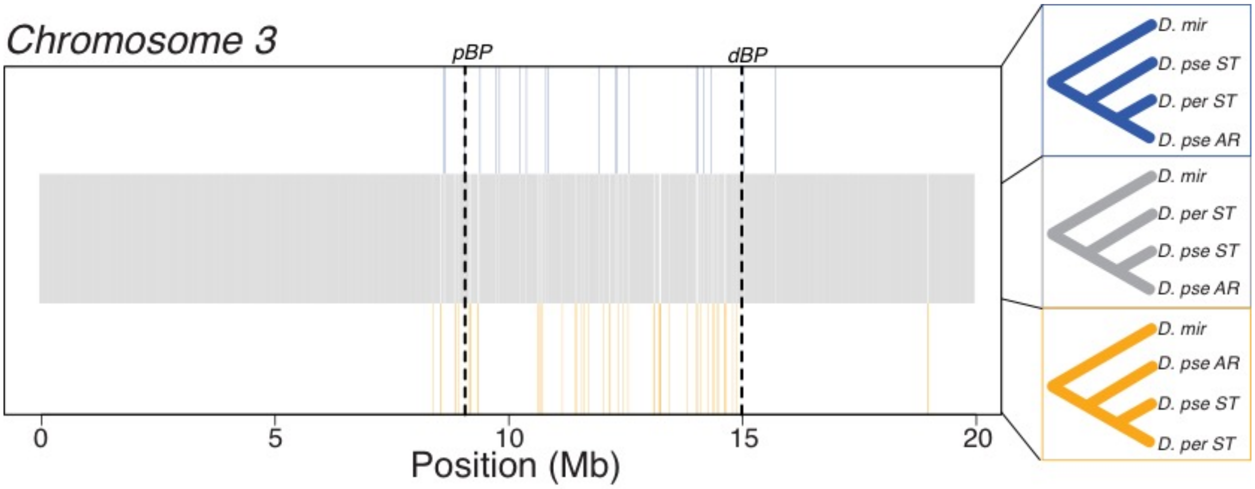

Supplemental Figure 4: **Introgression of the D*. pseudoobscura ST* arrangement into a *D. persimilis* genetic background.** Despite 15 generations of marker-assisted backcrossing, all hybrid males that carry the *D. pseudoobscura XR* material in an otherwise *D. persimilis* genetic background are sterile. These results indicate that the chromosome-level gene exchange must have happened before the evolution of hybrid incompatibilities on this chromosome arm.

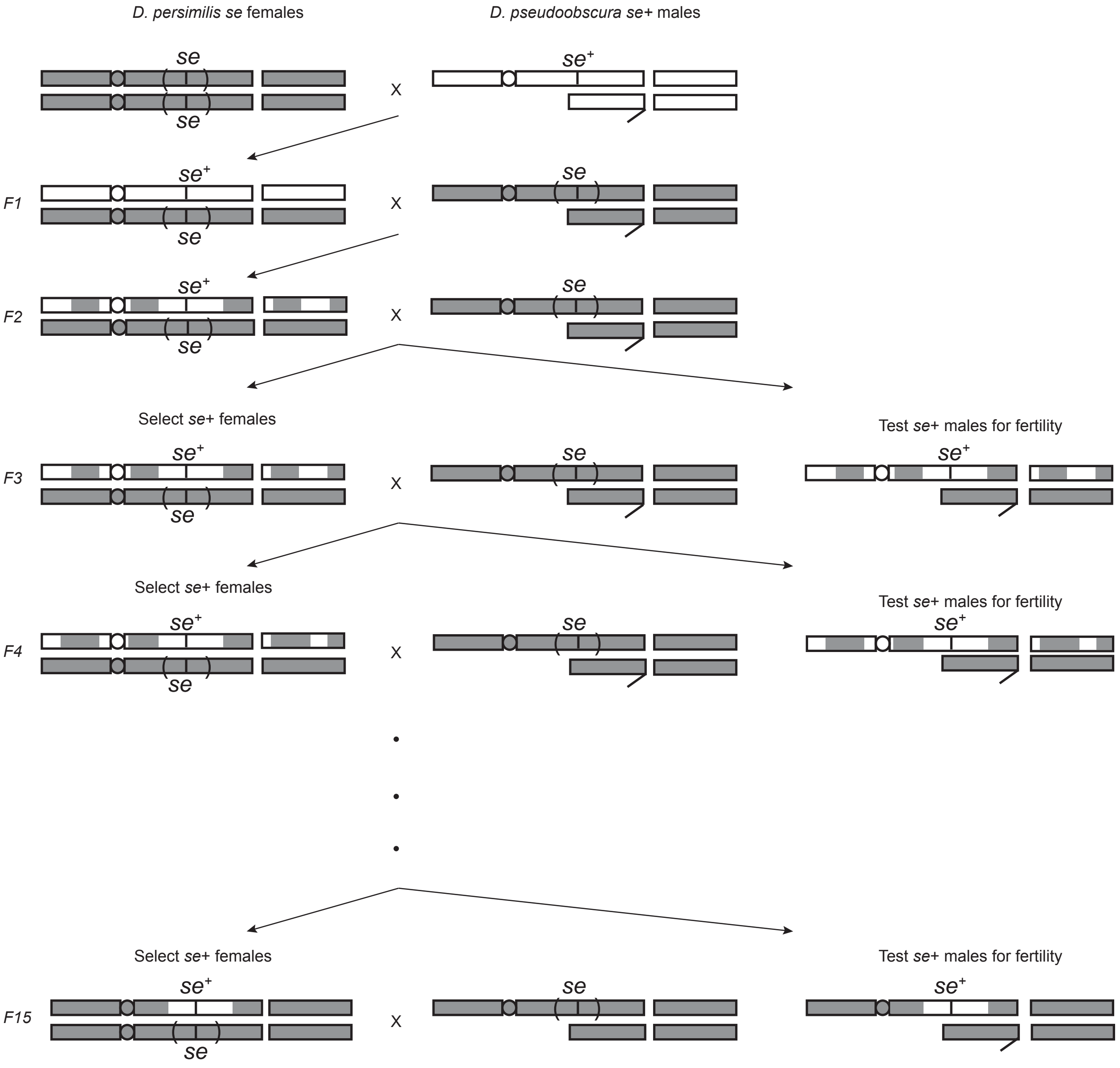

Supplemental Figure 5: Divergence in sliding windows across chromosomes. Smoothing splines are shown for divergence measured as relative node depth (RND) in 10kb windows across chromosomes XR (A), XL (B), and 2 (C). The different colors for each line indicate the taxa pair RND is estimated for, with the key in the legend. Colored dots represent individual windows that are in the top 1% of RND values genome-wide and are considered outliers. Black vertical lines indicate the locations of inversion breakpoints on each chromosome. The insets on XR show a close-up view of RND estimated around the proximal and distal inversion breakpoints ± 250 kb.

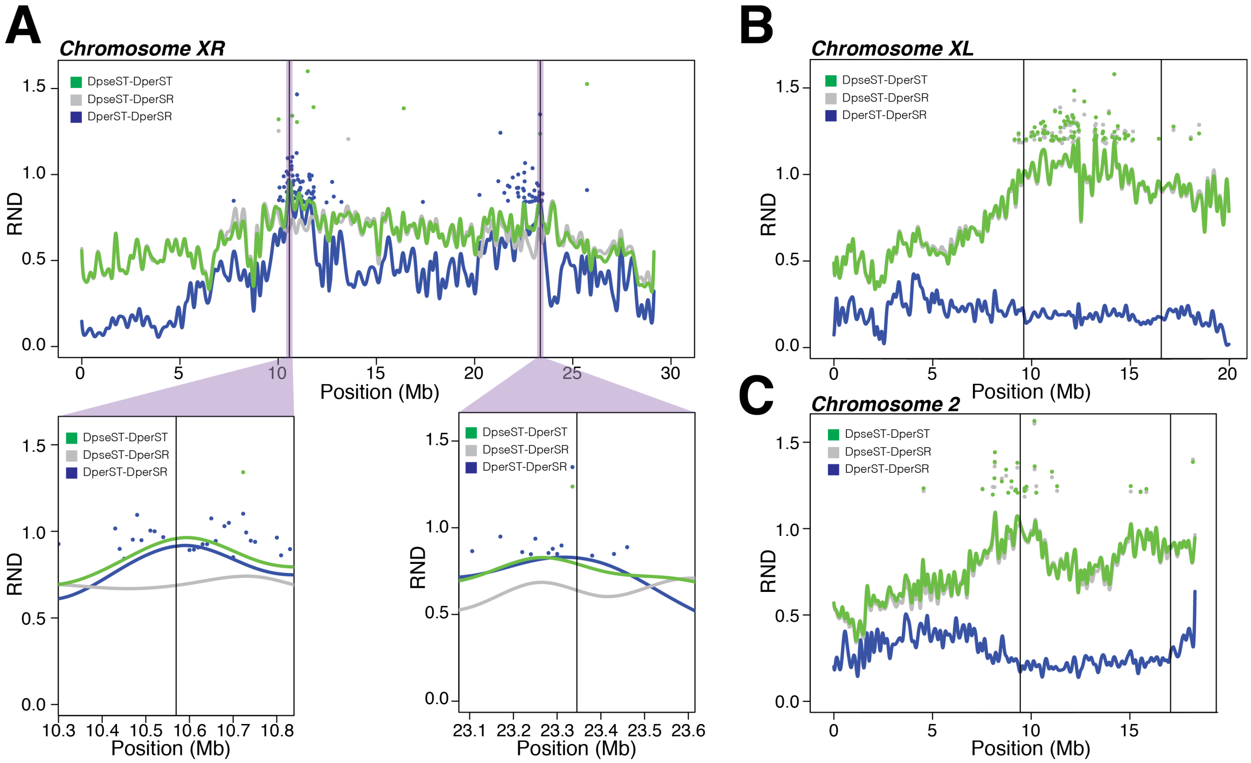

Supplemental Figure 5. Isolation with initial migration model. The width of the bars are proportional to the population sizes and the heights of bars indicate time using the maximum likelihood approach of Costa and Wilkinson-Herbots (2017). The ancestral population for each set of data is indicated by a single colored bar that splits into two subpopulations at time t_0. From t_0 to t_1 (V) the populations diverge in allopatry with the estimated levels of gene flow (M; in units of number of migrants per generation). At time t_1, the populations no longer exchange genes among the subpopulations. The vertical white bars are the confidence intervals for time t_0 and t_1. The collinear region represents species divergence, while XR, 2, and XL represent the divergence of fixed inversion differences between *D. pseudoobscura* and *D. persimilis*.

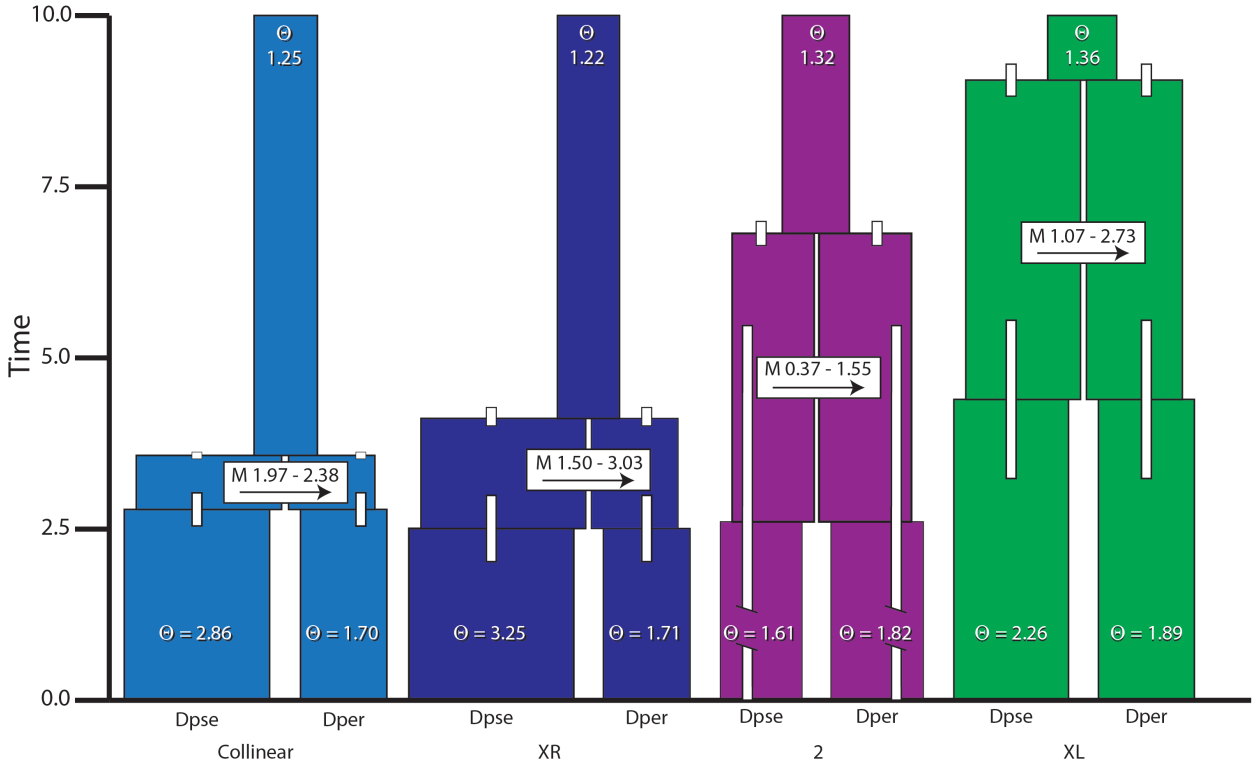

Supplemental Table 1: *D. pseudoosbcura* reference alignment statistics

Statistic	***D. pse ST***	***D. per ST***	***D. per SR***	***D. mir***
Total Reads	139337556	242743626	215111160	49649299
Mapped Reads	133512488	223244577	165073083	45556023
% Mapped	95.82	91.97	76.74	91.53
Mean Coverage	82.72	180.28	133.37	22.19
St. Dev. Coverage	236.03	666.46	538.60	60.15
% Reference Bases Covered (QUAL > 30, DP > 2)	96.49	95.48	95.45	89.77

Supplemental Table 2: *D. pseudoobcura* reference alignment statistics for each sample across all scaffolds

Scaffold	Mean Coverage	Length	% Covered	Covered (bp)	+ Reads	- Reads	Read GC	Median Coverage	St. Dev. Coverage
D. pseudoobscura ST									
2	83.9129	30819483	97.5788	30073269	13841040	13841340	0.4403	85	40.51
3	85.9616	19787792	97.5288	19298803	9058245	9057166	0.4549	86	62.53
XL_group3a	44.7765	2692213	96.4761	2597342	642496	643846	0.4455	43	67.92
XL_group3b	54.3022	388551	98.341	382105	112865	113304	0.4266	45	122.17
XL_group1e	42.4095	12541198	97.8198	12267770	2878802	2874461	0.4426	42	26.38
XL_group1a	45.7357	9148293	96.8705	8861993	2257478	2255726	0.4523	42	81.27
XR_group3a	40.5871	1469181	97.7692	1436406	324078	324013	0.4676	40	16.46
XR_group8	41.4123	9197557	97.3256	8951575	2056823	2057328	0.4571	41	20.03
XR_group6	42.6727	13333775	97.5782	13010864	3061992	3063993	0.4477	42	34.97
XR_group5	51.3656	740970	98.7634	731807	203154	203069	0.4408	44	49.9
4_group1	84.4553	5287126	98.1752	5190646	2402053	2400617	0.418	84	201.76
4_group2	81.5053	1235759	94.4519	1167198	534344	533721	0.4381	85	62.7
4_group5	80.7469	2439919	93.5238	2281905	1054607	1055036	0.4205	86	36.3
4_group3	83.7848	11685562	97.9635	11447582	5288757	5290633	0.438	84	38.18
4_group4	85.1283	6594820	96.8235	6385336	3016316	3013210	0.4307	86	58.84
D. persimilis ST									
2	164.977	30819483	97.0029	29895789	21218521	21207288	0.4407	167	188.31
3	172.5389	19787792	97.2785	19249261	14029962	14051884	0.4562	168	315.05
XL_group3a	98.7035	2692213	95.3536	2567123	1111653	1129452	0.4454	84	516.14
XL_group3b	127.3868	388551	96.5912	375306	205046	214191	0.428	87	646.91
XL_group1e	81.7047	12541198	96.4054	12090397	4416728	4410151	0.4437	81	101.25
XL_group1a	90.2694	9148293	95.7352	8758133	3551556	3546460	0.4523	81	236.71
XR_group3a	79.19	1469181	97.3737	1430596	486513	484643	0.4699	79	41.59
XR_group8	89.5757	9197557	96.9735	8919190	3458016	3455692	0.4542	80	325.56
XR_group6	85.1736	13333775	97.1807	12957860	4747154	4758411	0.4501	82	175.54
XR_group5	130.6743	740970	98.2654	728117	414469	400653	0.4468	86	578.93
4_group1	163.9504	5287126	98.0539	5184232	3610240	3612560	0.4204	167	214.27
4_group2	157.7765	1235759	94.0514	1162249	803902	801984	0.4409	165	130.01
4_group5	162.3184	2439919	93.3079	2276636	1642333	1646317	0.421	170	153.35
4_group3	167.8842	11685562	97.8139	11430105	8157166	8208579	0.4386	167	264.92
4_group4	174.5058	6594820	96.5778	6369135	4817701	4797167	0.4293	169	372.64
D. persimilis SR									
2	123.8174	30819483	97.066	29915249	15929350	15921833	0.4405	126	121.34
3	129.4046	19787792	97.3175	19256975	10524186	10542187	0.4561	126	200.77
XL_group3a	72.644	2692213	95.3993	2568353	819835	830492	0.4458	63	327.77
XL_group3b	91.683	388551	96.5912	375306	147636	153787	0.4273	65	411.43
XL_group1e	61.6822	12541198	96.4177	12091940	3337286	3330588	0.4433	61	71.76
XL_group1a	67.3117	9148293	95.7067	8755531	2650164	2642849	0.4521	61	156.07
XR_group3a	59.0851	1469181	97.0172	1425359	363124	361638	0.4681	60	26.84
XR_group8	67.4893	9197557	96.7935	8902635	2596009	2598475	0.4536	61	202.92
XR_group6	65.3798	13333775	96.9487	12926920	3641958	3650285	0.4502	62	125.81
XR_group5	108.0875	740970	98.2692	728145	341682	333001	0.4491	65	654.37
4_group1	122.9093	5287126	97.9889	5180798	2708350	2709811	0.4199	125	137.14
4_group2	121.8041	1235759	94.0568	1162315	619405	619577	0.4406	123	125.59
4_group5	122.0682	2439919	93.2785	2275919	1236125	1239514	0.4207	128	101.35
4_group3	125.9379	11685562	97.7793	11426058	6126711	6157243	0.4382	126	160.42
4_group4	130.2957	6594820	96.5311	6366054	3600688	3581896	0.4292	127	228.23
D. miranda									
2	18.7027	30819483	93.6237	28854340	4027647	4027315	0.4583	18	18.38
3	20.1168	19787792	94.4606	18691673	2779677	2785477	0.4691	19	26.53
XL_group3a	19.4302	2692213	92.6627	2494677	368119	368015	0.4623	18	32.81
XL_group3b	29.0281	388551	91.2452	354534	79627	79773	0.4578	19	47.92
XL_group1e	17.8448	12541198	92.6182	11615438	1585445	1585920	0.4625	17	21.8
XL_group1a	19.6939	9148293	91.9186	8408982	1275755	1276178	0.4701	18	44.68
XR_group3a	19.3349	1469181	93.2392	1369852	201744	200722	0.481	17	69.58
XR_group8	17.3782	9197557	94.1218	8656902	1121618	1119342	0.4722	17	11.37
XR_group6	18.1368	13333775	94.4924	12599410	1690148	1689856	0.4622	18	24.69
XR_group5	20.3789	740970	94.8644	702917	105668	105158	0.4537	19	21.71
4_group1	16.3376	5287126	90.9867	4810580	609460	610129	0.4451	16	16.38
4_group2	18.8393	1235759	89.9682	1111790	162242	162151	0.4547	18	20.14
4_group5	16.2932	2439919	88.1169	2149982	279051	278681	0.444	17	14.46
4_group3	17.8253	11685562	93.7048	10949928	1461704	1460165	0.4556	17	23.03
4_group4	17.2507	6594820	89.696	5915288	802786	803197	0.4491	17	22.14

Supplemental References:

1. Schaeffer SW, Goetting-Minesky MP, Kovacevic M, Peoples JR, Graybill JL, Miller JM, et al. Evolutionary genomics of inversions in Drosophila pseudoobscura: Evidence for epistasis. Proc Natl Acad Sci U S A. 2003;100: 8319–8324. doi:10.1073/pnas.1432900100

2. Dobzhansky TG, Epling CC. Contributions to the Genetics, Taxonomy, and Ecology of Drosophila Pseudoobscura and Its Relatives. Th. Dobzhansky and Carl Epling. - Washington 1944. 8°. Carnegie Institution of Washington; 1944.

3. Richards S, Liu Y, Bettencourt BR, Hradecky P, Letovsky S, Nielsen R, et al. Comparative genome sequencing of Drosophila pseudoobscura: Chromosomal, gene, and cis-element evolution. Genome Res. 2005;15: 1–18. doi:10.1101/gr.3059305

4. Schaeffer SW, Bhutkar A, McAllister BF, Matsuda M, Matzkin LM, O'Grady PM, et al. Polytene Chromosomal Maps of 11 Drosophila Species: The Order of Genomic Scaffolds Inferred From Genetic and Physical Maps. Genetics. 2008;179: 1601–1655. doi:10.1534/genetics.107.086074

5. Wallace AG, Detweiler D, Schaeffer SW. Evolutionary History of the Third Chromosome Gene Arrangements of Drosophila pseudoobscura Inferred from Inversion Breakpoints. Mol Biol Evol. 2011;28: 2219–2229. doi:10.1093/molbev/msr039
